# Low-frequency BOLD fluctuations demonstrate altered thalamocortical connectivity in diabetic neuropathic pain

**DOI:** 10.1186/1471-2202-10-138

**Published:** 2009-11-26

**Authors:** Franco Cauda, Katiuscia Sacco, Federico D'Agata, Sergio Duca, Dario Cocito, Giuliano Geminiani, Filippo Migliorati, Gianluca Isoardo

**Affiliations:** 1 CCS fMRI, Koelliker Hospital, Corso Galileo Ferraris, 251-255,10134, Turin, Italy; 2 Department of Psychology, University of Turin, Via Po, 14, 10123, Turin, Italy; 3 Department of Neuroscience, Azienda Ospedaliero Universitaria San Giovanni Battista, Via Cherasco,15, 10126, Turin, Italy; 4 Neurophysiology unit, Department of Neurosurgery, Azienda Sanitaria CTO, Via Zuretti, 29, 10126, Turin, Italy

## Abstract

**Background:**

In this paper we explored thalamocortical functional connectivity in a group of eight patients suffering from peripheral neuropathic pain (diabetic pain), and compared it with that of a group of healthy subjects. We hypothesized that functional interconnections between the thalamus and cortex can be altered after years of ongoing chronic neuropathic pain.

**Results:**

Functional connectivity was studied through a resting state functional magnetic resonance imaging (fMRI) paradigm: temporal correlations between predefined regions of interest (primary somatosensory cortex, ventral posterior lateral thalamic nucleus, medial dorsal thalamic nucleus) and the rest of the brain were systematically investigated. The patient group showed decreased resting state functional connectivity between the thalamus and the cortex.

**Conclusion:**

This supports the idea that chronic pain can alter thalamocortical connections causing a disruption of thalamic feedback, and the view of chronic pain as a thalamocortical dysrhythmia.

## Background

It is already known [1] that certain brain disorders, such as schizophrenia, Alzheimer's disease, autism, epilepsy and Parkinson's are associated with abnormal neural synchronization. This suggests a correlation between abnormalities in neuronal synchronization and cognitive dysfunctions, highlighting the importance of temporal coordination within the brain.

Some recent findings posed the possibility that pain can be related to the emergence of large-scale synchrony, occurring at two different frequency bands; a synchrony that can be enhanced or destroyed by attention and distraction [2]. These results seem to suggest that what matters most, in the case of cortical dynamics during pain, are not the oscillations themselves but their synchrony [3]. Pain seems to imply a "slowing down" of the cortical dynamics [4], and this dynamical aspect of the oscillations is consistent with Llinas's view of chronic pain as a thalamocortical dysrhythmia [5], where the slow rhythm is produced by a disruption of thalamocortical feedback. Indeed, experimental animal models [6] illustrated that, after induced chronic pain, the thalamocortical somatosensory loop shows important synaptic re-organization. A growing number of studies have examined the effects of attention on pain and its neural correlates (for a review see: [7-10]); in particular, experimental pain can decrease cognitive performance and task-related activity [11-13]. Although not completely understood, the cognitive processes underlying pain perception have been shown to be impaired in patients suffering from chronic pain [13-19]. Moreover several studies have reported anatomic, functional and biochemical changes in the thalamic region in patients suffering from chronic neuropathic pain suggesting that regions of the thalamus which have lost their normal somatosensory input or that show alterations in the metabolic balance can contain neurons which exhibit abnormal spontaneous and evoked activity and that electrical stimulation of these regions can produce the sensation of burning dysesthesia [7,20-24]. More specifically Iadarola and colleagues [25] using positron emission tomography found a unilateral decrease in thalamic activity in patients with chronic neuropathic pain suggesting that this condition can cause functional alterations in thalamic pain processing circuits.

The recent discovery of low-frequency band fluctuations (LFBF) in the blood oxygenation level dependent (BOLD) signal (for a review see [26]), identified during passive, resting state functional magnetic resonance imaging (fMRI) paradigms [27-30], gave researchers a new tool to explore the brain's network dynamics and pathology-related changes. The concept of functional connectivity, introduced by Karl Friston [31] as "The temporal correlations across cortical regions" could represent an index of brain function [31,32]. Intrinsic functional brain connectivity, revealed by low-frequency spontaneous signal fluctuations in fMRI signal time courses, has received increasing attention in the neuroscience community. Functionally organized systems were first described by Biswal and colleagues [33]; such resting state networks have been subsequently identified in various systems within the cerebral cortex, and related to specific types of sensory, motor and cognitive functions (for a review see [34]), suggesting the existence of several resting state networks in the human brain [27,35]. Recently, it has been demonstrated that resting state functional connectivity (rsFC) patterns are not artefactually produced by aliasing of cardiac and respiratory cycles, but are instead localized in the gray matter and are likely related to ongoing neuronal activity [36]. Indeed, the BOLD signal within the default mode network (DMN) cannot be explained by cardiac variation rate effects alone and is possibly related to some form of cognitive neuronal processing [27,37-39]. Moreover, it has been demonstrated [27] that these functionally organized systems display BOLD signal changes comparable to task-related BOLD changes (up to 3%) and that these networks are consistent across individuals and stable across repeated sessions.

Unfortunately, despite its central role in the functioning of the cerebral cortex [40,41], little is known about the participation of the thalamus in the brain's intrinsic activity. Occasionally, thalamocortical patterns of connectivity have been observed in resting state studies [34-36,42-46], but few attempts have been made to systematically characterize thalamic nuclei on the basis of spontaneous resting state activity or to explore the effect of pathologies on thalamic rsFC. To the best of our knowledge, only one paper has focused its attention on thalamocortical resting connectivity in normal subjects: Zhang and colleagues studied the intrinsic functional relations between human cerebral cortex and the thalamus [47] finding strong relationships between specific thalamic areas and the cortex. They also defined thalamic boundaries that may serve as a functional atlas for localizing functional areas of the thalamus. In the field of brain pathologies, Welsh et al. [48] used rsFC to show that thalamocortical functional connectivity is altered in schizophrenia; Laureys et al. [49] found that there is a restoration of thalamocortical connectivity after recovery from persistent vegetative state; White et al. [50] evidenced a disruption of functional interactions within neural networks involving the thalamus and cerebral cortex after general-anesthetic-induced unconsciousness; and Mizuno et al. [51] discovered a partially enhanced thalamocortical functional connectivity in autism. On the basis of these results and those of our previous work showing a general disruption of the rsFC in neuropathic pain [52], we hypothesized that thalamocortical reverberation can also be altered after years of ongoing chronic neuropathic pain. Thus, in the present work we investigated the temporal correlations between predefined regions of interest (ROI) in the thalamic nuclei and in the somatosensory cortex (S1) and the rest of the brain, using the method of functional connectivity. This network analysis approach [53] considers the role of a set of brain regions working in unison, assuming that the time courses of interconnected areas show higher correlations [54]. We compared a group of patients suffering from diabetic neuropathic pain with age- and gender-matched healthy controls.

## Methods

Eight right-handed consecutive patients suffering from peripheral NP (diabetic pain) (four women and four men; age range = 51-78, mean age = 61 years) were enrolled from our multidisciplinary pain unit (tab. s2). All patients underwent a complete neurological and psychological examination as well as standard MR brain scanning by an experienced neuroradiologist (SD) to exclude structural/white-matter abnormalities on anatomical MR-images. Patients suffering from significant psychiatric disorders were excluded. All patients were assessed using standardized pain scales (visual analog scale - VAS, numerical rating scale - NRS, McGill Pain Questionnaire MPQ - Italian version). The spontaneous component of their pain syndrome was evaluated on the MPQ checklist. VAS readings were obtained from their clinical records both the day before and on the day of the study to check the stability of the level of pain (in tab. s2 we reported the value for the day of the study). In all cases pain was restricted to the bilateral lower limbs. Duration of pain was >2 years in all cases. Patients were washed out of their medications at least one month before imaging (opioids or cannabinoids were never administered). At the time of scanning, pain intensity had reached pre-treatment levels. Maximum care was taken to avoid situations that could actually trigger evoked pain during the imaging sessions. Eight age- and gender-matched right-handed healthy volunteers (four women and four men; age range = 47-79, mean age = 59 years) acted as controls. None suffered from any neurological or psychiatric disorders, including chronic pain of any kind, nor had a history of drug or alcohol abuse. None were on medications known to alter brain activity. All females participating in the study were menopausal. All subjects gave their informed written consent, in line with the Declaration of Helsinki, and the study was approved by the local ethics committee.

### Task and image acquisition

All subjects were instructed simply to keep their eyes closed, think of nothing in particular, and not to fall asleep. After the scanning session, participants were asked if they had fallen asleep during the scan; subjects who provided a positive or dubious answer were excluded from the study.

Data acquisition was performed on a 1.5 Tesla INTERA™ scanner (Philips Medical Systems) with a SENSE high-field, high resolution (MRIDC) head coil optimized for functional imaging. Resting state functional -weighted images were acquired using echoplanar (EPI) sequences, with a repetition time (TR) of 2000 ms, an echo time (TE) of 50 ms and a 90° flip angle. The acquisition matrix was 64 × 64, the field of view (FoV) 200 mm. A total of 200 volumes were acquired; each volume consisted of 19 axial slices, parallel to the anterior-posterior (AC-PC) commissure; slice thickness was 4.5 mm with a 0.5 mm gap. Two scans were added at the beginning of functional scanning and the data discarded to reach a steady-state magnetization before acquiring the experimental data. In the same session, a set of three-dimensional high-resolution *T*_1_-weighted structural images was acquired for each participant. This data-set was acquired using a Fast Field Echo (FFE) sequence, with a TR of 25 ms, ultra-short TE and a 30° flip angle. The acquisition matrix was 256 × 256, the field of view (FoV) 256 mm. The set consisted of 160 contiguous sagittal images covering the whole brain. In-plane resolution was 1 × 1 mm^2 ^and slice thickness 1 mm (voxel of 1 × 1 × 1 mm^3^).

### Data analysis

BOLD imaging data were analyzed using the BrainVoyager QX software (Brain Innovation, Maastricht, Holland). Functional images were preprocessed to reduce artifacts [55] following pre-processing steps: 1) slice scan time correction, using a sinc interpolation algorithm; 2) 3D motion correction: all volumes were aligned spatially to the first volume by rigid body transformations, using a trilinear interpolation algorithm; 3) spatial smoothing using a Gaussian kernel of 6 mm FWHM for the primary sensory (S1) connectivity and 8 mm FWHM for the ventral posterior thalamic nucleus (VPL) connectivity. We used a minor spatial smoothing in the S1 connectivity data to improve the spatial resolution in the thalamic area; 4) temporal filters (i.e. linear trend removal and band pass filter of 0.01-0.1) to reduce low frequency fluctuation in the blood oxygen level dependent (BOLD) signal before the functional connectivity analysis [28,33].

After pre-processing, a series of steps were followed in order to allow for the precise anatomical location of brain activity to facilitate inter-subject averaging. First, each subject's slice-based functional scan was co-registered on his/her 3D high-resolution structural scan. Second, the 3D structural data-set of each subject was transformed into Talairach space [56]: the cerebrum was translated and rotated into the anterior-posterior commissure plane and then the borders of the cerebrum were identified. Third, using the anatomo-functional coregistration matrix and the determined Talairach reference points, the functional time course of each subject was transformed into Talairach space and the volume time course created.

### ROI selection

To obtain the S1 ROI we extracted the location of the peak voxel from a localizer study conducted on eight healthy subjects (random effect analysis, thresholded at q<0.05 corrected with false discovery rate [FDR] [57]) and compared it with anatomic atlases and anatomic landmarks; over the bilateral peak voxel locations we designed a cubic ROI of 5 × 5 × 5 mm^3 ^(coordinates: -43, -26, 49; -46, -22, 49). The localizer study (following Moore et al. [58]) was a blocked design with interleaved epochs of tactile stimulation (brushing) and rest. For determination of thalamocortical connectivity, a seed region in the ventral posterior lateral nucleus (VPL) was determined using an anatomic atlas: we calculated the center of gravity of all voxels comprised in the VPL as mapped in the Talairach Daemon database [59]. Left and right 5 × 5 × 5 mm^3 ^seed ROIs were drawn over these two points (coordinates: 20, -20, 5; -18, -20, 5). In addition, since in the S1 rsFC a relevant area within the medial dorsal thalamic nucleus (MDN) was found to be less connected, we also computed a seed region in the bilateral MDN as determined using an anatomic atlas: we calculated the center of gravity of all voxels comprised in the MDN as mapped in the Talairach Daemon database [59]. Left and right 5 × 5 × 5 mm^3 ^seed ROIs were drawn over these two points (coordinates: +6, -22, 8; -6, -22, 8). All ROIs are listed in tab. s3.

Additional analyses were conducted using alternative ROIs, i.e. ROIs which were moved from the original location in the rostral, lateral, ventral, medial and caudal directions (3 mm), reduced (3 × 3 × 3 voxels) and increased (8 × 8 × 8 voxels) in dimensions. We found high similarity between the resulting maps and those obtained using the original ROIs: probabilistic maps showed high overlapping between the original and the alternative connectivity maps (see Additional file 1: figure S19). A probabilistic map describes the relative frequency (expressed in percent in our cases) in a sample of different maps with which a specific voxel or area is significantly activated.

### Functional connectivity analysis

The first step in all functional connectivity (FC) analyses was to extract BOLD time courses from each ROI by averaging over voxels within each region. Several nuisance covariates were included in the analyses to control for the effects of physiological processes (such as fluctuations related to cardiac and respiratory cycles) [60,61] and motion. Specifically, we included nine additional covariates that modeled nuisance signals from white matter (WM) and cerebrospinal fluid (CSF), global signal (GS) [62], as well as six motion parameters (three rotations and three translations). Additional file 1: Figure s21 shows the overlapping (probabilistic map) of voxels correlated with GS. All seed-based predictors were z-normalized. Temporal autocorrelation correction [63] was used to eliminate the autocorrelation induced by the temporal lowpass filter. Seed ROI-driven FC maps were computed on a voxelwise basis for each previously selected region. Individual participant multiple regression analysis was carried out using the general linear model (GLM) [64] and resulted in a t-based map (SPMt) corrected for multiple comparisons with FDR [57] (q<0.05, cluster threshold >5 voxels in the native resolution).

### Group statistical map

Random effect group-level analyses and group comparisons controlling for age and gender effects were conducted using the ANCOVA analysis tool implemented in BrainVoyager QX. Corrections for multiple comparisons were carried out using the FDR [57] (q<0.05, cluster threshold >5 voxels in the native resolution).

Given the variable age in our group, we conducted random effects analyses of covariance (ANCOVAs) to assess the potential effect of this variable on the spatially specific activations revealed in the preceding analyses. To test for significant group-related differences, direct voxelwise group comparisons were performed using group-level contrasts. These contrasts computed the voxelwise statistical significance of mean group differences in FC, and produced FDR thresholded maps of those voxels that showed significant differences between the two groups. For MDN and VPL connectivity the resulting maps were then projected on a 3D representation of the brain using the BrainVoyager QX 3D cortical tool.

To evaluate the spatial consistency of functional connectivity patterns across subjects we computed probabilistic maps, that were calculated separately for each ROI-generated network.

### Pairwise correlations between the four a priori ROIs

For each subject and each ROI, we extracted the time course at each voxel. Using a home-made SPSS script, we computed the mean time course for each ROI. For each subject we performed pairwise correlations between the ROIs' mean time courses, in order to assess rsFC. In particular, within each hemisphere, we assessed the rsFC between the thalamus (VPL) and S1; we also assessed the rsFC between each area and its homologous area in the opposite hemisphere (left and right thalamus; left and right S1). Parametric analyses (t-tests for independent samples) were used at the group level to determine the statistical significance of the difference between patients and controls on each paired correlation.

### Lateralization

In connectivity comparison between the pain group and the control group we calculated the lateralization (percent of left or right voxels over the total) of the portion of functionally connected Brodmann areas (see additional methods).

### Voxel distance calculations

We calculated the Euclidean distance between the center of gravity of the three pairs of ROI (MDN left and right, VPL left and right, S1 left and right) and every other voxel that reached significance in the thresholded Z-score map of positive FC (cluster significance: p < 0.05). The Euclidian distance between two voxels P = (*p*_*x*_, *p*_*y*_, *p*_*z*_) and Q = (*q*_*x*_, *q*_*y*_, *q*_*z*_) was computed using the formula . We computed the number of significant voxels at specific distances (from 0 to 140 mm in 4-mm bins) for each individual, then we rebinned these values in three ranges thus comprised: short- (<40 mm), medium- (40-80 mm) and long- (>80 mm) distance connections. Finally we calculated group means and compared them using a simple t-test.

## Results

The aim of the study was to compare the resting state functional connectivity (rsFC) in the thalamus and in the primary somatosensory cortex between normal and pain subjects.

Although we used age as a covariate we further inspected possible age effects on the rsFC maps performing a correlational analysis between each subject-specific ROI-generated map and the relative age using the ANCOVA analysis tool implemented in BrainVoyager QX. We found no significant correlation between them (q<0.05 FDR corrected). Movement was assessed by summing the deviations (three translations plus three rotations at a radius of 50 mm) used to compensate for head motion within fMRI. The quantity reported here is head movement root mean square averaged over subjects. This quantity was 0.30 ± 0.09 mm (mean ± standard deviation) for patients and 0.28 ± 0.10 mm (mean ± standard deviation) for age-matched controls. Patients vs. controls comparisons were not significantly different (p = 0.84). Hence, it is unlikely that movement can account for the observed differences in connectivity between the two samples.

### Functional connectivity of S1

**Figure 1:**
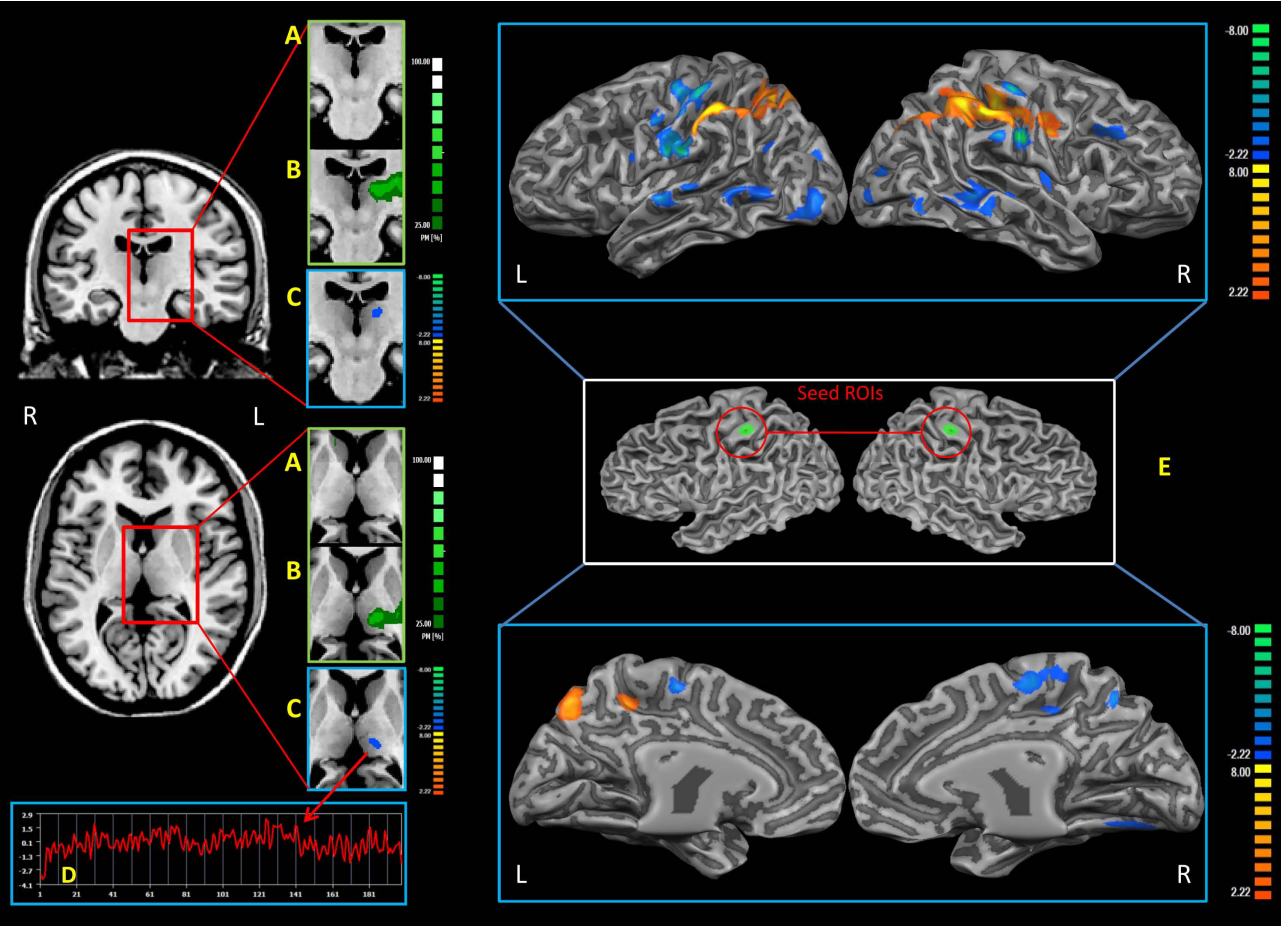
**S1 resting state connectivity analysis**. A: Probabilistic maps of the S1 rsFC thalamic projection relative to pain subjects. B: Probabilistic maps of the S1 rsFC thalamic projection relative to healthy subjects. C: Group comparison of the S1 rsFC thalamic projection (Two sample t-test q<0.05 FDR-corrected, minimum cluster dimension K>5 voxels in the native resolution). D: Example of the time course of a healthy subject. E: Group comparison of the S1 rsFC brain projection (Two sample t-test q<0.05 FDR-corrected, minimum cluster dimension K>5 voxels in the native resolution). Right middle panel represents the Seed ROI. Colors from green to white indicate an increasing spatial overlapping probability (%) (single subject correlation maps before probabilistic map creation thresholded at q<0.05 FDR-corrected, minimum cluster dimension K>5 voxels in the native resolution). Colors from red to yellow indicate an increased connectivity in the pain group; colors from blue to green indicate a reduced connectivity in the pain group. All rsFC maps were projected on 3D representations of the brain using BrainVoyager QX.

Comparing the pain and healthy groups (see fig. 1 and also Additional file 1: s10, s22), patients showed a decrease in rsFC between S1 and the left thalamus, including several different nuclei, such as the MDN, laterodorsal (LDN), and pulvinar. The MDN was the most represented nucleus, with 76 voxels found to be less connected with respect to the control group (see Additional file 1: Figure s9). As regards the connectivity between S1 and the rest of the cortex, in the patient group we found several areas of reduced connectivity (Additional file 1: Figure. S12; Table. s4, s6) localized in the bilateral middle temporal gyrus, superior temporal gyrus, pre/post central gyrus, paracentral lobule, middle frontal gyrus, middle occipital gyrus, and cingulated cortex; right putamen, cuneus, fusiform gyrus, lingual gyrus and declive; left superior frontal gyrus and inferior occipital gyrus. The wider areas were in the postcentral gyrus (BA 2, 3; Left Lateralization L = 64%), middle temporal gyrus (BA 22, 21; L = 54%) and superior temporal gyrus (BA 22, 21; L = 72%).

An increase of rsFC in the patient group (Additional file 1: fig. s13; tab. s5, s6) was found bilaterally in the inferior and superior parietal lobule, precuneus, postcentral gyrus (this increased connectivity area is more posterior and less extended compared to the decreased connectivity area in the postcentral gyrus described above) and in the right precentral gyrus. The wider areas were in the inferior parietal lobule (BA 40, L = 69%) and the precuneus (BA 7, Right Lateralization R = 70%).

To assess the spatial consistency of seed-generated S1 correlation patterns across subjects we computed spatial probabilistic maps (see fig. 1). Overall, both groups showed a high overlapping of subjects' maps; more specifically, 62% of the healthy subjects showed S1 connections in the left thalamus and 50% in the right thalamus, while only 12% of the patients showed areas of connectivity in the left thalamus and 0% in the right thalamus.

The healthy group showed no significant differences, compared to the pain group, in mean, medium and long-distance connectivity, but only in short-distance connectivity (Additional file 1: Figure. s18). In all cases patients had fewer connected voxels.

### Functional connectivity of VPL

**Figure 2:**
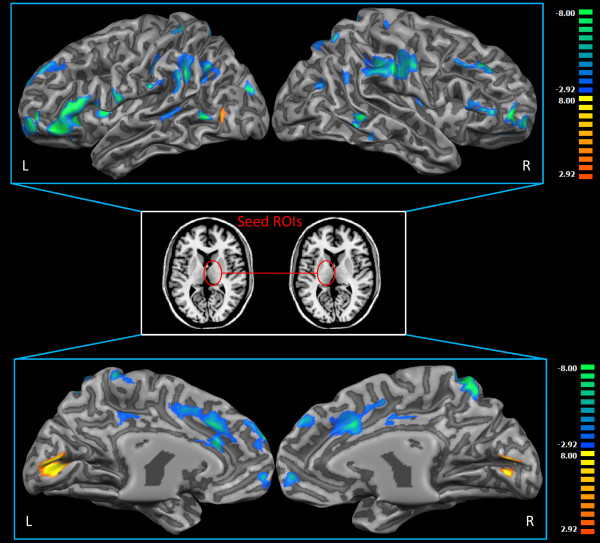
**VPL resting state connectivity analysis**. Group comparison of the VPL rsFC brain projection (Two sample t-test q<0.05 FDR-corrected, minimum cluster dimension K>5 voxels in the native resolution). Middle panel represents the Seed ROI. Colors from red to yellow indicate an increased connectivity in the pain group; colors from blue to green indicate a reduced connectivity in the pain group. All rsFC maps were projected on 3D representations of the brain using BrainVoyager QX.

Patients showed a generalized decrease in rsFC (see fig. 2, Additional file 1: s11, s14, s23; tab. s7, s9) between VPL and the bilateral pre/postcentral gyrus, inferior parietal lobule, superior parietal lobule, supramarginal gyrus, precuneus, insula, middle frontal gyrus, inferior frontal gyrus, superior frontal gyrus, medial frontal gyrus and cingulated cortex; right caudate and middle temporal gyrus; left putamen, pulvinar and MDN. The wider areas were in the middle frontal gyrus (BA 10, 9, 46, 6; L = 45%), inferior frontal gyrus (BA 45, 46, 47, 13; L = 50%) and superior frontal gyrus (BA 10, 9, 6, 8; L = 68%).

An increase of rsFC in the patient group (see fig. 2, Additional file 1: s15; tab. s8, s9) was found in the bilateral cuneus, bilateral posterior cingulated cortex and bilateral lingual gyrus. The wider area was in the lingual gyrus (BA 18, 19; L = 91%).

To assess the spatial consistency of seed-generated VPL correlation patterns across subjects we computed spatial probabilistic maps. Overall, both groups showed a high overlapping of subjects' maps; more specifically, 75% of the healthy subjects showed VPL connections in the bilateral pre/postcentral gyrus and 66% in the cingulated cortex, while only 62% of the patients showed areas of connectivity in the bilateral pre/postcentral gyrus and 37% in the cingulated cortex.

The healthy group showed no significant differences, compared to the pain group, in mean, medium and long-distance connectivity but only in short-distance connectivity (Additional file 1: fig. s18) and in all cases patients had fewer connected voxels.

### Functional connectivity of MDN

**Figure 3:**
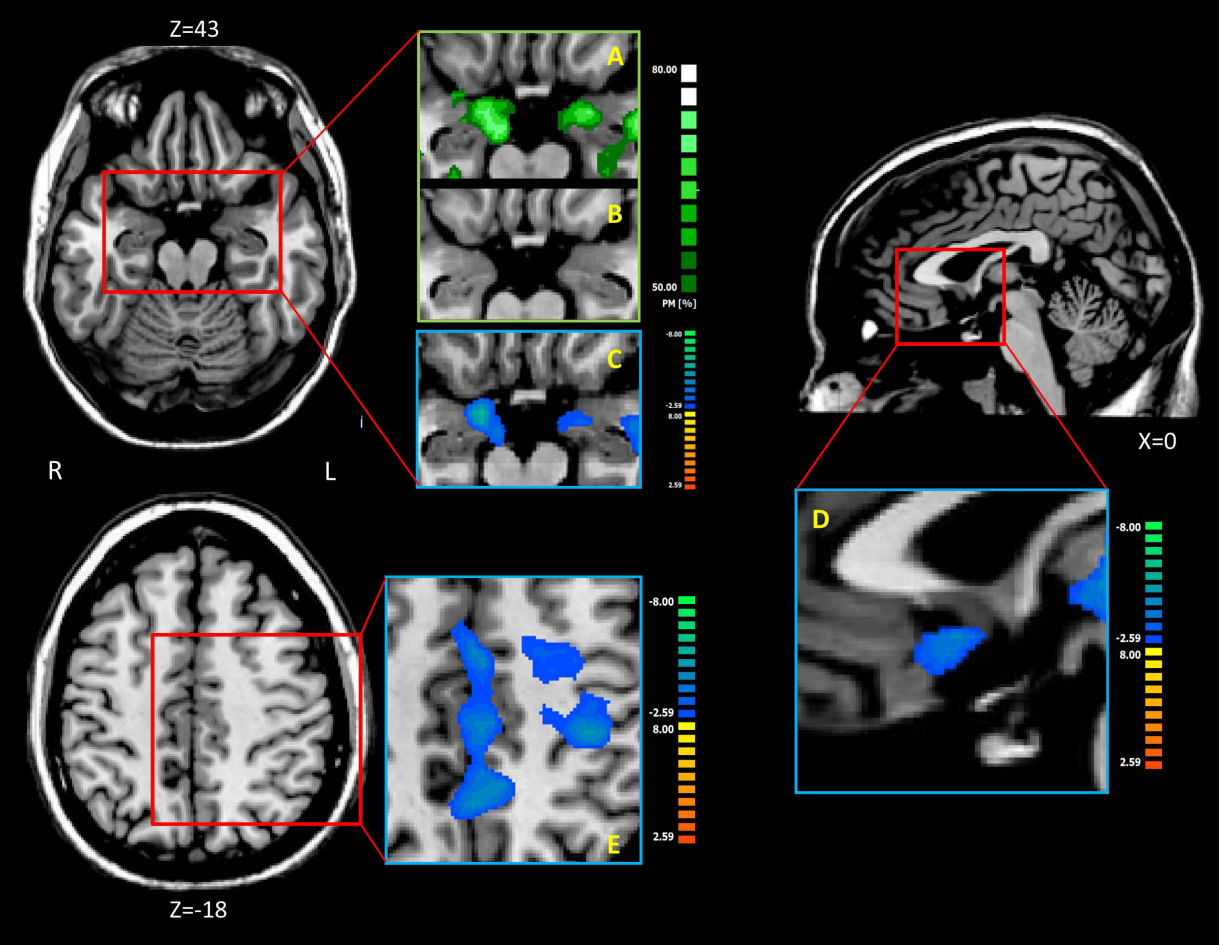
**MDN resting state connectivity analysis**. A: Probabilistic maps of the MDN rsFC Amygdale projection relative to healthy subjects. B: Probabilistic maps of the MDN rsFC Amygdale projection relative to pain subjects. C: Group comparison of the MDN rsFC Amygdale projection. D: Group comparison of the MDN rsFC Subgenual ACC projection. E: Group comparison of the MDN rsFC S1 projection. Colors from green to white indicate an increasing spatial overlapping probability (%) (single subject correlation maps before probability map creation thresholded at q<0.05 FDR-corrected, minimum cluster dimension K>5 voxels in the native resolution). Group comparison with two sample t-test, q<0.05 FDR-corrected, minimum cluster dimension K>5 voxels in the native resolution; colors from red to yellow indicate an increased connectivity in the pain group; colors from blue to green indicate a reduced connectivity in the pain group. All rsFC maps were projected on 3D representation of the brain using BrainVoyager QX.

Patients showed a generalized decrease of rsFC (see fig. 3, Additional file 1: s16, s23; tab s10, s12) in the bilateral frontal gyrus (inferior, middle, superior), pre/postcentral gyrus, paracentral lobule, precuneus, cingulated cortex, insula, superior temporal gyrus, inferior parietal lobule, pulvinar, parahippocampal gyrus, amygdale, cerebellar tonsil; right pyramis, uvula, nodule; left supramarginal gyrus, VPL, VLN, globus pallidus, caudate. The wider areas were in the left superior temporal gyrus (BA 41, 13, 22, 42; L = 100%) and in the cuneus (BA 30, 18, 23; L = 65%).

An increase of rsFC in the patient group (see fig. 3, Additional file 1: s17; tab s11, s12) was found in the bilateral cuneus, posterior cingulated, lingual gyrus; left insula and transverse temporal gyrus. The wider areas were in the inferior frontal gyrus (BA 47, 45, 46, 44; L = 75%), middle frontal gyrus (BA 10, 6, 11, 47; L = 79%) and in the precuneus (BA 7, 31; L = 68%).

To assess the spatial consistency of seed-generated MDN correlation patterns across subjects we computed spatial probabilistic maps. Overall, as with S1 and VPL connectivity, both groups showed a high overlapping of the subject specific maps (see fig. 3), 75% of the healthy subjects showed an area connected to MDN in the left amygdala and 74% in the right amygdala while just 20% and 22% of patients showed areas of connectivity (left and right amygdala respectively).

The healthy group showed no significant difference, compared to the pain group, in mean, short, medium and long-distance connectivity (Additional file 1: fig. s18). Patients always had fewer connected voxels but they were not statistically significant.

### Pairwise correlations

**Figure 4:**
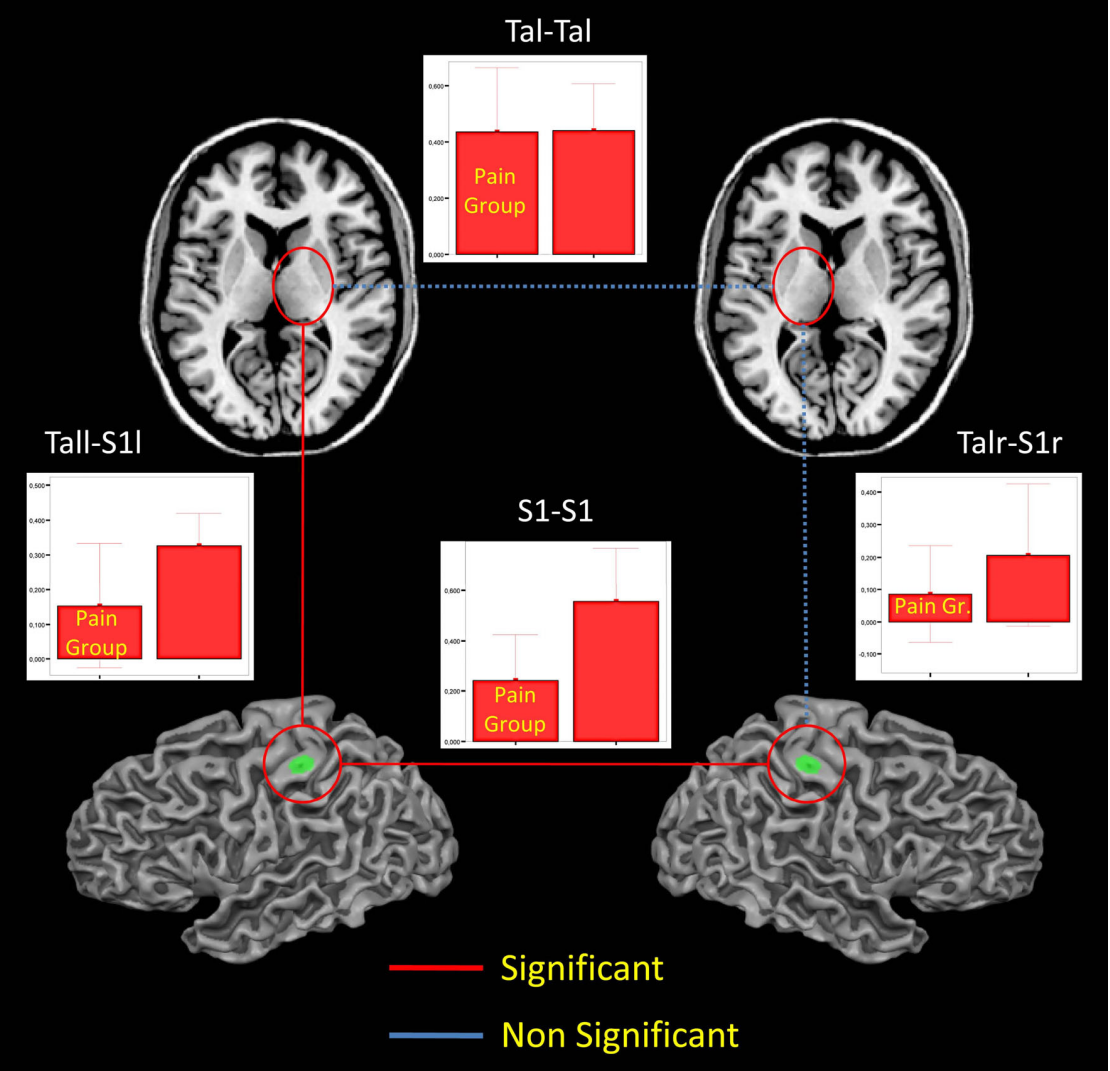
**Pairwise correlations**. Mean correlations and standard deviations for each pair of investigated ROIs in the patient and control groups. Red lines indicate statistically relevant differences in rsFC between the two groups, blue dotted lines indicate no statistically relevant differences.

Results of pairwise correlations are illustrated in fig. 4. Significant differences emerged between patients and controls in rsFC between left and right S1 (t = 2.670; p = 0.09), and, within the left hemisphere, between S1 and VPL (t = 2.039; p = 0.03). No significant differences emerged between the two thalami (VPL) and between right S1 and right VPL.

## Discussion

The methodology we used is particularly suited to analyze brain networks as a whole so we will focus the discussion on the general increase or reduction in thalamic network connectivity rather than on changes in specific cortical areas. The main finding of the present study is decreased thalamocortical connectivity in patients with chronic painful diabetic polyneuropathy. More specifically we found a reduced synchrony between S1 and some of the thalamic nuclei involved in pain processing. Exploring specifically the VPL and MDN connectivity we demonstrated that VPL has a reduced synchrony with sensorymotor, frontal, cingulated and parietal areas while MDN more with sensory and emotional processing areas.

Chronic neuropathic pain, as the result of a predominantly small fiber sensory polyneuropathy, is a frequent complication of diabetes, and patients with impaired glucose tolerance may also suffer from pain [65]. Thus, pain may be a complication of diabetic neuropathy even in the early stages. Differently from previous studies on chronic back pain [66], we dealt with the effect of both deafferentation and neuropathic pain on cerebral functions. There is growing evidence, stemming from both neurophysiological and functional imaging studies, that lesions of the peripheral nerve may sustain plastic changes of the central nervous system, at both cortical and subcortical level. For instance, tremor and dystonia were sometimes observed after a peripheral nerve trauma as well as complex regional pain syndrome [67]. Ischemic nerve block is associated with decreased intracortical inhibition evaluated by transcranial magnetic stimulation [68]. Indeed, modification of cerebral and spinal activity may also be related to pain [69]. For instance, withdrawal reflexes as a result of modification of spinal interneuronal activity may be modulated by heterotopic painful stimuli [70], and painful electrical stimulation of the median nerve may modulate cortical excitability [71]. However, long-term modifications induced by chronic pain on brain function are still a matter of debate [72], and this uncertainty may depend on the different types of neuropsychological and functional imaging protocols employed so far. Pain is characterized by both a spontaneous and an induced dimension (i.e. allodynia). While functional imaging studies based on the analysis of brain activation after painful stimuli seem to be more suitable for evaluating the induced pain mechanism, analysis of resting state or default mode of brain function is an appealing method of evaluating modification of brain activity induced by ongoing spontaneous pain [66]. In everyday neurology patients with chronic neuropathic pain are commonly found to experience the spontaneous painful sensation more when awake and at rest than during daily activities. In a previous study on the same cohort of patients [52] we observed that during rest there is a reduced default mode network [73] connectivity of bilateral primary sensorimotor areas and cingulated cortex, as well as left temporal and occipital cortices; on the other hand, there was increased connectivity in the left dorsolateral prefrontal cortex and posterior parietal cortex, (corresponding to area 7) lateral thalamus and bilateral insula. These results point toward an opposite modification of connectivity in the cingulated cortex and the insulae which are well-recognized parts of the so-called pain matrix, a network of brain areas functionally linked and activated during processing of painful thermal stimuli in healthy subjects [2]. The increased connectivity in the left dorsolateral prefrontal cortex may be coupled with the decreased connectivity in the anterior cingulated cortex, since the former area exerts an inhibitory control on the latter as well as on the medial thalamus [74]. Thus, the reduced connectivity of the medial thalamus observed in the present study is also in keeping with the findings of our previous work. The inhibitory control of the dorsolateral prefrontal cortex on the medial-thalamus-anterior cingulated cortex loop accounts for the reduction of perceived unpleasantness [74] associated with allodynia in patients with sensitized skin. Thus, in patients who suffer frequently from allodynia, such as those affected by diabetic polyneuropathy, a modulation of the left dorsolateral prefrontal cortex-anterior cingulated cortex-medial thalamus loop could take place constitutively. This is further suggested by the findings that show a decreased regional blood flow at rest in the anterior cingulated cortex in patients with chronic pain.

Another possible interpretation is that the reduced connectivity of the anterior cingulated cortex in our patients as well as others with chronic pain is related to mood modulation. This is suggested by the results of stimulation of the motor cortex in patients with chronic intractable pain [75]: PET studies on patients with decreased pain showed an increase in cerebral blood flow in the anterior cingulate cortex and orbitofrontal cortex. The anterior cingulated cortex and medial thalamus are parts of both the system analyzing the affective dimension of pain as well as the reward system encompassing the ventral striatum. The anterior cingulate cortex has connection with both the medial prefrontal cortex and the amygdala. Indeed the present study also found the amygdala to have reduced connectivity to the medial thalamus. The amygdala is implicated in the modulation of perception of emotional stimuli, particularly the enhanced perception of salient stimuli and memory of emotional experience. It also has a role in behavior control related to emotional stimuli, indeed patients with lesions of this structure have blunting of emotional and fear perceptions [76].

Taken together these results may suggest that in our patients there is an increased connectivity of cerebral areas related to inhibitory control of pain (left dorsolateral prefrontal cortex), and a reduced connectivity of areas related to modulation of emotional response to stimuli (amygdale, anterior cingulated cortex, caudate). Although this study did not include any formal evaluation of mood, patients with diabetic painful polyneuropathy frequently suffer from depression as well as sleep disturbances [77], which interfere with quality of life.

Another interesting observation on connectivity of cortical areas pertains to the increased connectivity of the primary sensory cortex with the posterior as well as the inferior parietal cortex. The latter result may be interpreted on the basis of the known role of the posterior parietal cortex in sequential motor performance, the so-called "internal controller" function matching efferent copy of motor command with sensory feedback [78]. In the presence of an altered sensory feedback, an increased connectivity between the primary sensory cortex and the "internal controller" may represent an adaptive plastic change to overcome difficulties in complex movements. These observations may also be related to the known role of the precuneus in episodic memory as well as self-awareness[79]. It is notable that in our patients precuneus connectivity with S1 was increased, but its connectivity with the medial thalamus, which is the major subcortical target of the precuneus, was decreased. Although the present study did not include a formal evaluation of memory and self-awareness, it is tempting to speculate that in patients with continuous pain there may be a rearrangement of the structures that are involved in episodic memory and this may represent a protective adaptation. This is also in keeping with the reduced connectivity of the amygdale, which is involved in the memory of emotional information [76].

Focusing more closely on the reduced connectivity of the VPL and MDN nuclei of the thalamus observed in the present study, this is consistent with the observation reported above and with a recent study in which subjects with diabetic neuropathic pain had significant reduction in brain metabolites (NAA) in the thalamus compared with the no-pain group [80]. A PET study on complex regional pain syndrome showed that the thalamus seemed to be hypoactive at least in the chronic stage of the disease [81]. This correlates with the increased regional blood flow in the thalamus after motor cortex stimulation for intractable pain (Garda Larrea e Peyron 2007). It has been suggested that activation of the thalamus is a crucial event leading to an eventual, albeit insufficient pain relieving effect.

Modulation of thalamic activity in pain patients should also be interpreted in the light of electrophysiological studies.

Pain, in healthy controls, has the unique capability of disrupting the idling electroencephalogram (EEG) rhythm on a large-scale spatial dimension (i.e. both alpha on the occipital areas and mu-rhythm on the sensorimotor areas [82]). On the contrary, it induces gamma oscillations in the sensory cortex [83]. Since thalamus activity is increased during wakefulness with closed eyes, that is the typical state in which alpha rhythm predominates, it is conceivable that a continuous or long-standing stimulus that disrupts a typical idling rhythm such as pain, may result in the deactivation of the thalamus. This correlates with the reduced large-scale connectivity of all the thalamic regions considered in our study. Activation of the direct and indirect pathways of the basal ganglia has the effect of inducing both excitation of some cortical neuronal populations and inhibition of surrounding neurons, in order to focus the neuronal output on the desired behavior or sensation [84]. This type of neuronal central excitation/surrounding inhibition is mirrored by the already known event-related de-synchronization surrounded by hyper-synchronization [85] and is particularly applicable to the frontal-subcortical circuit encompassing the medial dorsal thalamus, which also involves the striatum and the pallidum [86]. A reduced connectivity of the thalamus may underlie a disruption of these mechanisms and thus may constitute a true dysrhythmic syndrome, as hypothesized by Llinas [87]. Neuropathic pain, as well as other neurological disorders such as Parkinson's disease, is actually considered part of the dysrhythmic syndrome. Thus, combining the results of the present and our previous study, it is conceivable to hypothesize that a dysrhythmic syndrome may be present in diabetic patients with persistent neuropathic pain.

The relative role of pain vs. deafferentation in the induction of the alteration of thalamus and cortex connectivity in our patients remains unresolved. However, some evidence points toward a crucial role of neuropathic pain. Firstly, the selective reduction of activity in the medial dorsal thalamus and ventrolateral nucleus is consistent with a specific pain-related phenomenon since both nuclei are part of the respectively medial and lateral spinothalamic tract. Secondly, even if these patients were affected by polyneuropathy with electrophysiological evidence of involvement of A beta fibers (data not shown), the influence of these alterations on brain function may be hypothesized to be more evident during tasks and may be reflected by a disruption of the so-called optimal feedback control circuit which is made by the primary sensorimotor, posterior parietal and premotor cortices [[88], Wolpert cited above]. Thirdly, Baliki et al. [66] recently demonstrated that a disruption of default mode of brain function occurs in chronic back pain. Thus, our results seem to be related to a common mechanism of disruption of default mode and dysrhythmia of the brain which is shared by various conditions associated with neuropathic pain. However, a possible compromise between the importance of deafferentation vs. that of pain in the modulation of cerebral cortex may be suggested by the theory of the introceptive system [89]. This system comprises the A delta and C fibers, regardless of their fine specificity (thermoceptive, nociceptive) or site of innervation (somatic vs. visceral), the lamin spinal I neurons, reticular nuclei of the brainstem such as the A1 and A5-7 monoaminargic group and parabrachial nucleus, as well as the medial dorsal and ventrobasal thalamus and insular and anterior cingulated cortex. This system is implicated in the evaluation of the homeostatic control of the entire body and is not only involved in sensory perception, but also modulates sympathetic responses and it is also hypothesized to modulate more complex motor behavior. From this perspective, a profound modulation of thalamic connectivity at rest as reported herein is conceivable.

Taking this view the left lateralization of the S1-thalamic connectivity reduction seems difficult to explain, however as shown by the pairwise correlation (see fig. 4) the right S1-Thalamic connectivity, while not significant, is similar to that of the left and just below the threshold: we hypothesize that with a bigger group of patients we would also find the right connectivity to be significantly reduced.

From this perspective the distinction between brain function modulation induced by pain and by deafferentation may be less meaningful.

## Conclusion

In summary, exploring thalamocortical functional connectivity in a group of eight patients suffering from peripheral neuropathic pain (diabetic pain), and compared it with that of a group of healthy subjects we found a reduced synchrony between S1 and some of the thalamic nuclei involved in pain processing. Studying specifically the VPL and MDN connectivity we demonstrated that VPL has a reduced synchrony with sensorymotor, frontal, cingulated and parietal areas while MDN more with sensory and emotional processing areas. This supports the idea that chronic pain can alter thalamocortical connections causing a disruption of thalamic feedback, and the view of chronic pain as a thalamocortical dysrhythmia.

### Methodological Consideration

This study enrolled only a limited number of patients so the inference on a general population may be partly impaired. However, as mentioned earlier, our results are consistent with previous results.

The distinction between modulation induced by pain or by deafferentation, although less meaningful in the light of the theory of the introceptive system, should be analyzed in further studies comparing patients with diabetic neuropathy with and without pain.

A major point remains unresolved: is the reduced connectivity of the medial dorsal thalamus an adaptive or misadaptive plastic change? Although it continues to be a matter of debate, the medial dorsal thalamus together with other limbic structures (fornix) seems to play an important role both in retaining and in re-learning encoded information [90,91]. Therefore, further studies should address the issue of learning and memory as well as depression in diabetic patients with painful polyneuropathy and their relationship with alteration of default mode of brain function.

## List of Abbreviations

BOLD: blood oxygenation level dependent; CSF: cerebrospinal fluid; GS: global signal; LFBF: low-frequency band fluctuations; MDN: medial dorsal nucleus; NP: neuropathic pain; ROI: region of interest; FC: functional connectivity; rsFC: resting state functional connectivity; S1: primary somatosensory area; VPL: ventral posterior lateral nucleus; WM: white matter.

## Authors' contributions

Experiments conceived and designed by: CF SK DS CD. Experiments performed by: CF SK DS CD. Data analyzed by: CF SK DF FM. Reagents/materials/analysis tools contributed by: CF SK DS CD FM DF GG. Paper written by: CF GI SK DF. All authors read and approved the final manuscript.

## Supplementary Material

Additional file 1 **Supplementary online material**. additional methods, results, figures and tables. Click here for file
