# Supplementary material for: Low-frequency BOLD fluctuations demonstrate altered thalamocortical connectivity in diabetic neuropathic pain
Source: BMC Neurosci. 2009 Nov 26;10:138. doi: 10.1186/1471-2202-10-138 (PMC2789078; doi:10.1186/1471-2202-10-138)
Supplement: Additional file 1 — Supplementary online material. additional methods, results, figures and tables. [file 1471-2202-10-138-S1.DOC]

**Supplementary online material**

**Additional Methods**

We created a Maltab® script (version 7.0) for voxel of interest (VOI) analysis to improve result summarization efficacy. We saved two atlases in .mat format, using AFNI data collection (http://afni.nimh.nih.gov/afni/ doc/misc/afni_ttatlas), in which the atlases are arrays of 140x172x120 voxels with a 1x1x1 mm^3^ resolution. The first was created for Gyrus classification of the normalized brain in the Talairach space (fig. s1) and the second for Brodmann Areas (fig. s2).

**Figure s1: Gyrus AFNI Template**

**
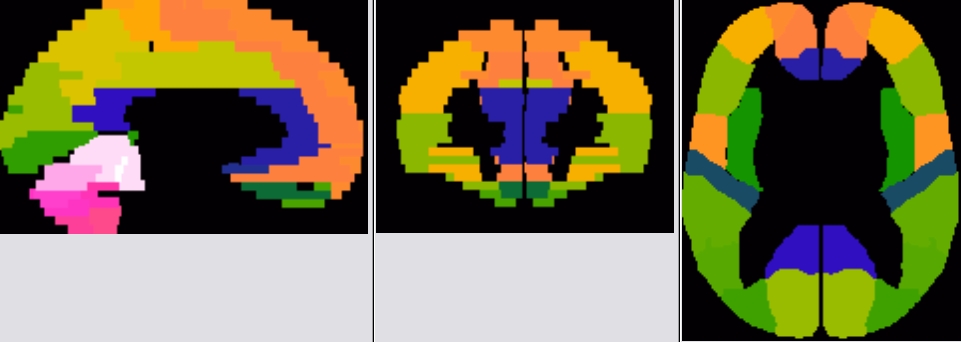
**

Three orthogonal slices of the template showing different Gyri in different colors

**Figure s2: Brodmann Areas AFNI Template**

**
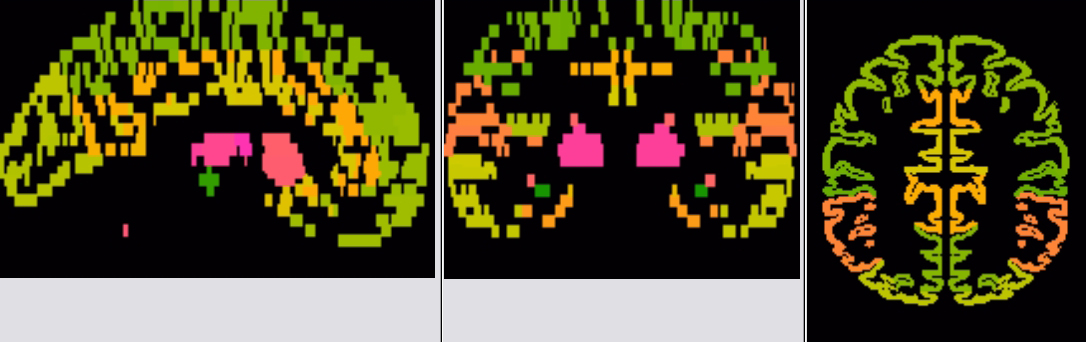
**

Three orthogonal slices of the template showing different BAs in different colors

Next we created other atlases forcing the classification of null voxels on the basis of a simple algorithm: if the absolute majority (>0.5) of nearest voxels (see fig. s3 bottom left) belong to a category we assigned that voxel to it. Using the algorithm iteratively we saved eight more atlases (called R1, R2, R3, R4) with an increasing number of classified voxels, but also with some image degradation (see fig. s3).

The inputs of the script were .voi files saved from BrainVoyager QX volume maps. These files contained samples of statistically significant voxels, divided into clusters. The script can compare these with the selected atlases to produce the outputs described in the following paragraphs.

**Figure s3: Brodmann Area AFNI R1 Template**

**
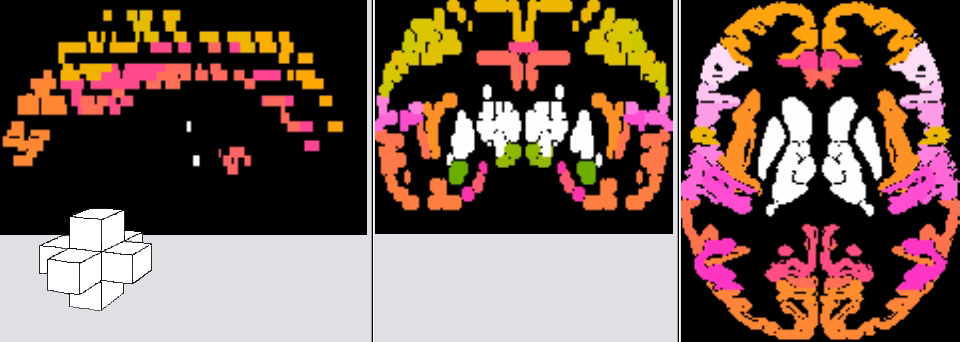
**

Three orthogonal slices of the template showing different BAs in different colors after near voxel forced classification

***Gyrus Output***

In the Gyrus output mode the script produces three graphs using the selected AFNI Gyrus atlas.

- The percentage of active voxels divided by the cerebral gyri (see fig. s4 on the left), the procedure stops at a fixed threshold of the total number of voxels (e.g. with a 5% threshold the script graphs the gyri that contain a number of active voxels greater than 5% of total active voxels, see fig. s4)
- The lateralization percent of the gyri that overcome the threshold (see fig. s4 on the right).
- The number of active voxels of the supra-threshold areas as a function of the gyrus and of the Talairach coordinate X (left to right, see fig. s5).


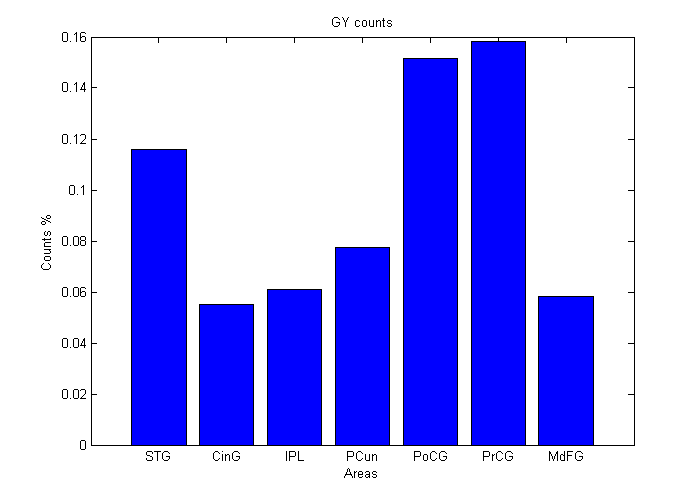

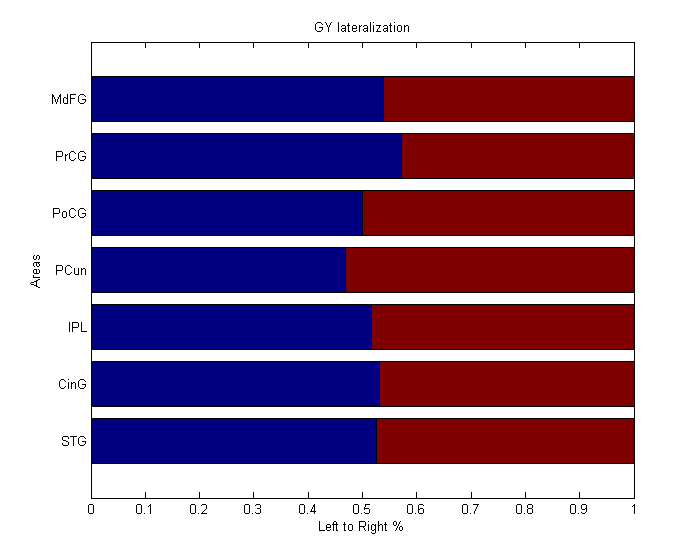
**Figure s4: Gyrus Output 1 & 2**

Gyrus counts percent (left) and gyrus lateralization (right)

**Figure s5: Gyrus Output 3**


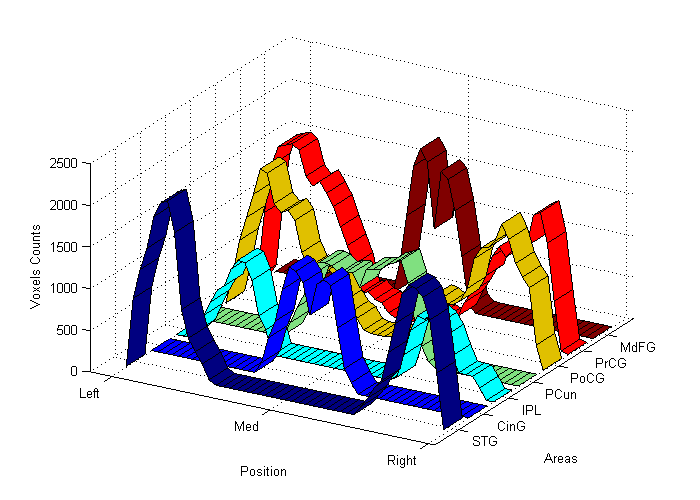
Gyrus counts as a function of areas and lateralization

The possible abbreviations for the output figures are: PCC = Posterior Cingulate; ACC = Anterior Cingulate; SbCG = Subcallosal Gyrus; TTG = Transverse Temporal Gyrus; Unc = Uncus; RG = Rectal Gyrus; FG = Fusiform Gyrus; IOG = Inferior Occipital Gyrus; ITG = Inferior Temporal Gyrus; Ins = Insula; PaHG = Parahippocampal Gyrus; LG = Lingual Gyrus; MOG = Middle Occipital Gyrus; OrG = Orbital Gyrus; MTG = Middle Temporal Gyrus; STG = Superior Temporal Gyrus; SOG = Superior Occipital Gyrus; IFG = Inferior Frontal Gyrus; Cun = Cuneus; Ang = Angular Gyrus; SMG = Supramarginal Gyrus; CinG = Cingulate Gyrus; IPL = Inferior Parietal Lobule; Pcun = Precuneus; SPL = Superior Parietal Lobule; MFG = Middle Frontal Gyrus; PaCL = Paracentral Lobule; PoCG = Postcentral Gyrus; PrCG = Precentral Gyrus; SFG = Superior Frontal Gyrus; MdFG = Medial Frontal Gyrus; vUv = Uvula of Vermis; vPyr = Pyramis of Vermis; vTub = Tuber of Vermis; vDec = Declive of Vermis; vCul = Culmen of Vermis; Cton = Cerebellar Tonsil; SLun = Inferior Semi-Lunar Lobule; Fast = Fastigium; Dent = Dentate; Nod = Nodule; Uvu = Uvula; Pyr = Pyramis; Tub = Tuber; Dec = Declive; Cul = Culmen; Clin = Cerebellar Lingual.

***Brodmann Areas Output***

The Brodmann Areas mode produces the same three graphs described above using a BA AFNI atlas.

The possible abbreviations are: Hippo = Hippocampus; Amg = Amygdala; HyTH = Hypothalamus; SN = Substantia Nigra; CauTa = Caudate Tail; CauBo = Caudate Body; CauHd = Caudate Head; VAN = Ventral Anterior Nucleus; VPMN = Ventral Posterior Medial Nucleus; VPLN = Ventral Posterior Lateral Nucleus; MDN = Medial Dorsal Nucleus; LDN = Lateral Dorsal Nucleus; Pulv = Pulvinar; LPN = Lateral Posterior Nucleus; VLN = Ventral Lateral Nucleus; MN = Midline Nucleus; AN = Anterior Nucleus; MaBo = Mammillary Body; Md GP = Medial Globus Pallidus; Lt GP = Lateral Globus Pallidus; Put = Putamen; NAcc =Nucleus Accumbens; MGB = Medial Geniculum Body; LGB = Lateral Geniculum Body; SuTH = Subthalamic Nucleus; BA 1-47 = Brodmann Area 1-47.

***Activation Output***

In the Activation mode the voxels are assigned to one of three groups: cerebellar voxels (CRBL), subcortical voxels (SCTX) and cortical voxels (CRTX). The relative percents of the groups are graphed together with their lateralization (see fig. s6). The classification proceeds in a serial fashion starting with the AFNI Gyrus atlas (CRTX and CRBL) and ending the remaining voxels with the AFNI BA atlas. The unclassifiable voxels are excluded from the graph (i.e. sum of the three groups = 100%).

**Figure s6: Activation Output**

**
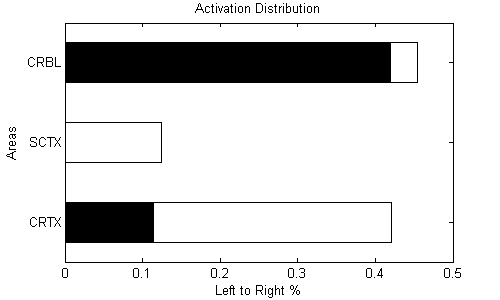
**

Percentages of cortical, subcortical and cerebellar total activation

***Subcortical Output***

In the Subcortical mode the output is a graph of the left and right fraction of the voxels that are active (i.e. 100% = all area voxels are activated) in some important non-cortical areas (see fig. s7).

**Figure s7: Subcortical Output**


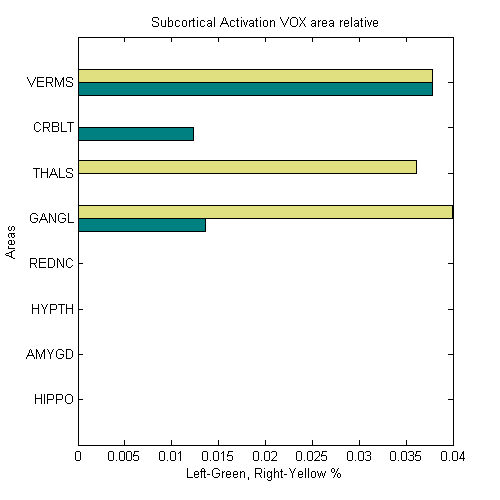
Fraction of active non-cortical area voxels

Abbreviations: VERMS = Vermis; CRBLT = Lateral Cerebellum; THALS = Thalamus; GANGL = Basal Ganglia; REDNC = Red Nucleus; HYPTH = Hypothalamus; AMYGD = Amygdala; HIPPO = Hippocampus.

***Segmentation Output***

In the Segmentation mode the voxels of the BA or Gyrus mode are saved in .voi or .img (analyze) format files (see fig. s8).

**Figure s8: Segmentation Output**

**
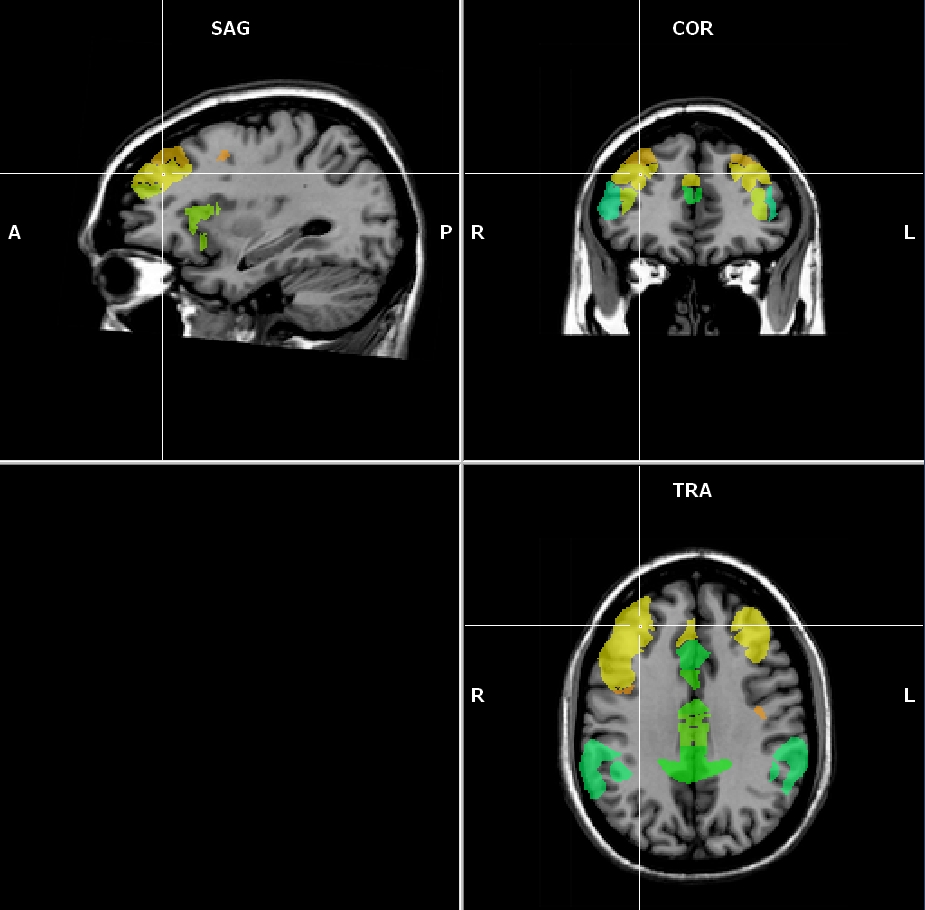

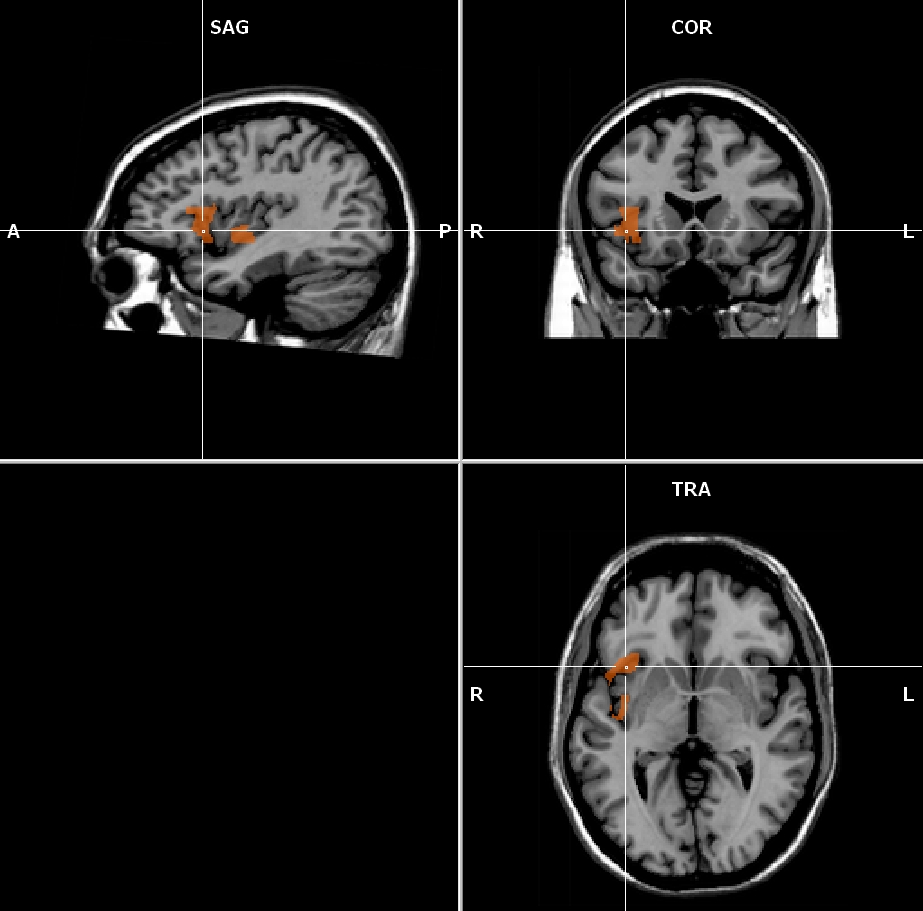
**

In different color voxels from different BAs (left) and insular active voxels subsample (right)

displayed by BrainVoyager QX

***Table Output***

In Table Mode the output is a .txt file with many rows (see tab. s1 for an example). Every row represents an area that meets some criteria: the number of active voxels included in the area surpasses a fixed fraction of total activation voxels (default = 5%) and/or the number of active voxels surpasses a fixed fraction of the total voxels of that area (default = 25%) and/or the number of active voxels surpasses a fixed absolute number of voxels in subcortical areas (default = 125) and the number of active voxels surpasses a minimum fixed absolute number of voxels (default = 100).

Every row contains the following data: name of the gyrus preceded by the left, right or bilateral attribute, number of active voxels, lateralization, Brodmann Areas of the voxels group in descending order.

The maximum number of Brodmann Areas written in the table is fixed (default = 4) so as not to obscure output readability.

The output can be of the total activation voxels or cluster by cluster also with the Tailarach's coordinates of peak activations including their gyrus and Brodmann Area specification.

The script and the atlases (in .mat or .img format) are available to anyone for examination or use. If interested, please send an email to federico.dagata@unito.it.

**Additional Results and Discussion**

We performed two additional analyses to further investigate whether the decreased cortico-thalamo-cortical connectivity between the pain group and the control group was related to the presence of chronic pain or was due to the direct effect of diabetes.

- First we calculated the difference of rsFC between pain and control groups of two more networks: the linguistic network, the performance of which is described as impaired in diabetic patients, and the auditory network, usually referred to as non-impaired by diabetes [1].

To do this first we took two symmetrical ROIs of 5x5x5 mm^3^ in the Broca’s area (centered in X = ±41, Y = +14, Z = 17) and two symmetrical ROIs of 5x5x5 mm^3^ in the primary auditory cortex (centered in X = ±46, Y = -26, Z = 12). The coordinates were chosen on the basis of the Brede Database (http://hendrix.ei.dtu.dk/services/jerne/brede/, see [2]).

Then we used the methods described in the article to produce the group statistical random effect group-level analysis maps. These maps (q<0.05 FDR-corrected, minimum cluster dimension >5 voxels in the native resolution) failed to show any significant differences (increased or decreased FC) between the two groups for both networks.

We also computed probabilistic maps of these two rsFC networks, which showed a very good spatial overlap for all sixteen subjects (see figure s20).

- Second, as suggested by a recent study [3] Global Signal (GS) regression has important implications for resting state studies attempting to compare conditions or groups: "if global signal regression is used in such studies, it is important to consider potential group differences in the distribution of the global signal to avoid misleading results". In our study those differences could, in theory, be related to the diabetic state so we investigated whether the GS fluctuations can account for differences between groups. We did this by contrasting the correlation between GS with all voxels within the brain of the pain group vs control group (two sample t-test, q<0.05 FDR corrected) finding no significant differences. We also computed the probabilistic map of all 16 subjects’ GS correlation (see figure s21). These GS-related patterns of connectivity are similar to those reported by Fox et al. [3] in their recent study.

All these findings point toward the hypothesis that the reduction in thalamocortical functional connectivity found in the pain group was more probably related to the long-lasting presence of pain than to an overall effect of diabetes. However, further studies comparing patients with diabetic neuropathy with and without pain are needed in order to provide a definitive answer to this question.

**Methodological considerations**

All voxelwise correlations are multivariate: we included all the seed ROIs in the multiple regression (GLM). With this method, we compute the statistical dependencies between two regions after removing the confounding effects of all other regions, hence providing data-driven measures that are closer to effective connectivity than bi-variate correlation. Of course, there still remains the possibility that a dependency detected by this method as significant is actually induced by the effect of a brain area that has not been considered in the analysis. Regarding the regions that have not been considered for this investigation, nothing can be said on their influence on the thalamocortical network explored in this work. What can be said, however, is that in the case of a lack of connection between two areas, the influence of a third area is little important.

**REFERENCES**

1. Kodl CT, Seaquist ER: **Cognitive dysfunction and diabetes mellitus**. *Endocr Rev* 2008, **29**(4):494-511.

2. Nielsen FA: **The Brede database: a small database for functional neuroimaging**. In *9th International Conference on Functional Mapping of the Human Brain* New York; 2003.

3. Fox MD, Zhang D, Snyder AZ, Raichle ME: **The Global Signal and Observed Anticorrelated Resting State Brain Networks**. *J Neurophysiol* 2009.

**Additional Figures**

**Figure s9: S1 rsFC group comparison 3D**

Two sample t-test q<0.05 FDR-corrected, minimum cluster dimension >5 voxels in the native resolution

Colors from red to yellow indicate an increased connectivity in the pain group

Colors from blue to green indicate a reduced connectivity in the pain group

Maps projected on the 3D renders of LDN, Pulvinar and MDN surfaces

**Fig s10: S1 rsFC group comparison 2D**

Two sample t-test q<0.05 FDR-corrected, minimum cluster dimension >5 voxels in the native resolution

Colors from red to yellow indicate an increased connectivity in the pain group

Colors from blue to green indicate a reduced connectivity in the pain group

Maps projected on the 2D slices at displayed Talairach’s coordinates

**Fig s11: VPL rsFC group comparison 2D**

Two sample t-test q<0.05 FDR-corrected, minimum cluster dimension >5 voxels in the native resolution

Colors from red to yellow indicate an increased connectivity in the pain group

Colors from blue to green indicate a reduced connectivity in the pain group

**Fig s12: Activation distribution of the S1rsFC increased connectivity**


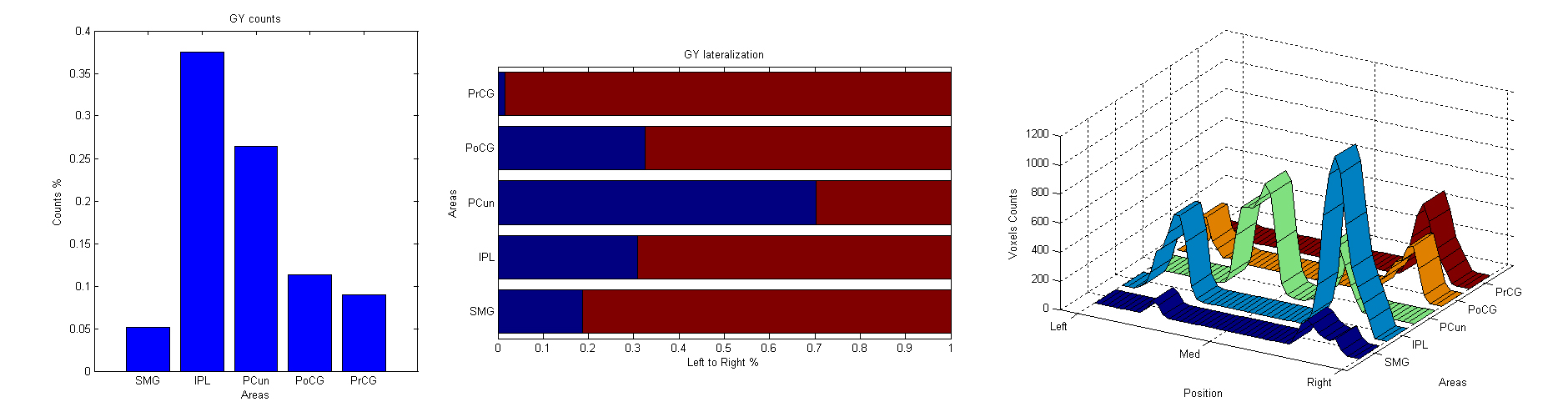


Most engaged gyri, from left to right: counts percent as function of areas, area lateralization (left blue, right red) and counts as function of area and X TAL coordinate; Abbreviations SMG = Supramarginal Gyrus, IPL = Inferior Parietal Lobule, PCun = Precuneus, PoCG = Postcentral Gyrus, PrCG = Precentral Gyrus; Two sample t-test q<0.05 FDR-corrected, minimum cluster dimension >5 voxels in the native resolution

**Fig s13: Activation distribution of the S1rsFC decreased connectivity**

**
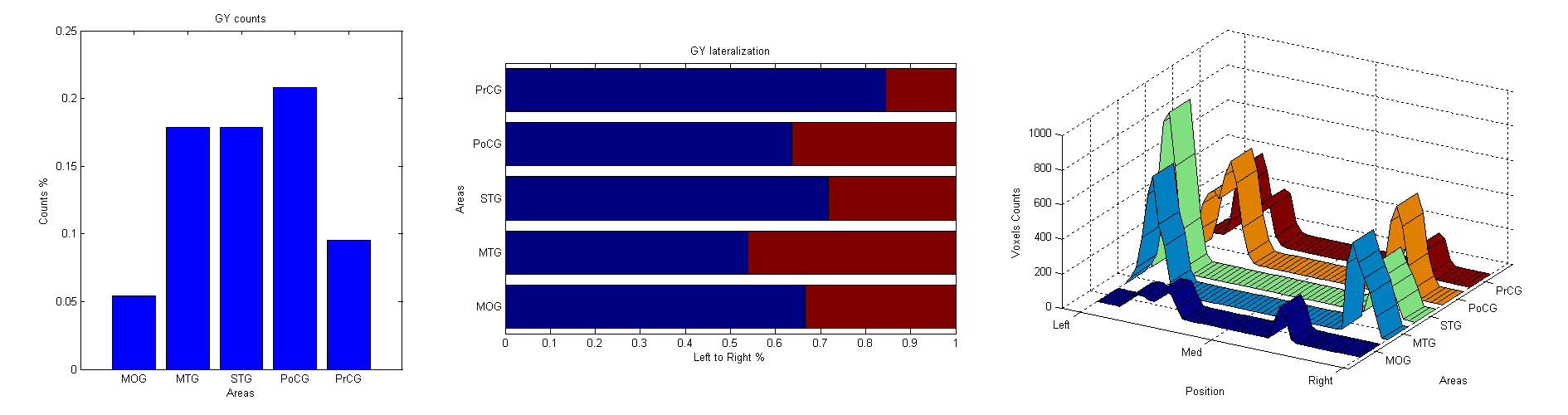
**

Most engaged gyri, from left to right: counts percent as function of areas, area lateralization (left blue, right red) and counts as function of area and X TAL coordinate; Abbreviations MOG = Middle Occipital Gyrus, MTG = Middle Temporal Gyrus, STG = Superior Temporal Gyrus, PoCG = Postcentral Gyrus, PrCG = Precentral Gyrus; Two sample t-test q<0.05 FDR-corrected, minimum cluster dimension >5 voxels in the native resolution

**Fig s14: Activation distribution of the VPL rsFC increased connectivity**

**
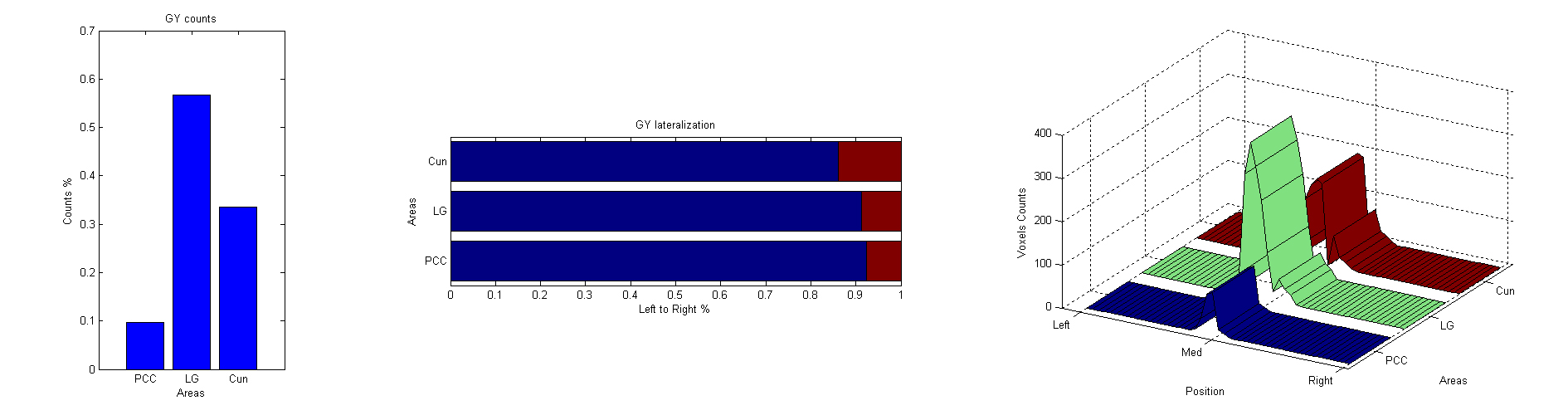
**

Most engaged gyri, from left to right: counts percent as function of areas, area lateralization (left blue, right red) and counts as function of area and X TAL coordinate; Abbreviations PCC = Posterior Cingulate Gyrus, LG = Lingual Gyrus, Cun = Cuneus; Two sample t-test q<0.05 FDR-corrected, minimum cluster dimension >5 voxels in the native resolution

**Fig s15: Activation distribution of the VPL rsFC decreased connectivity**

**
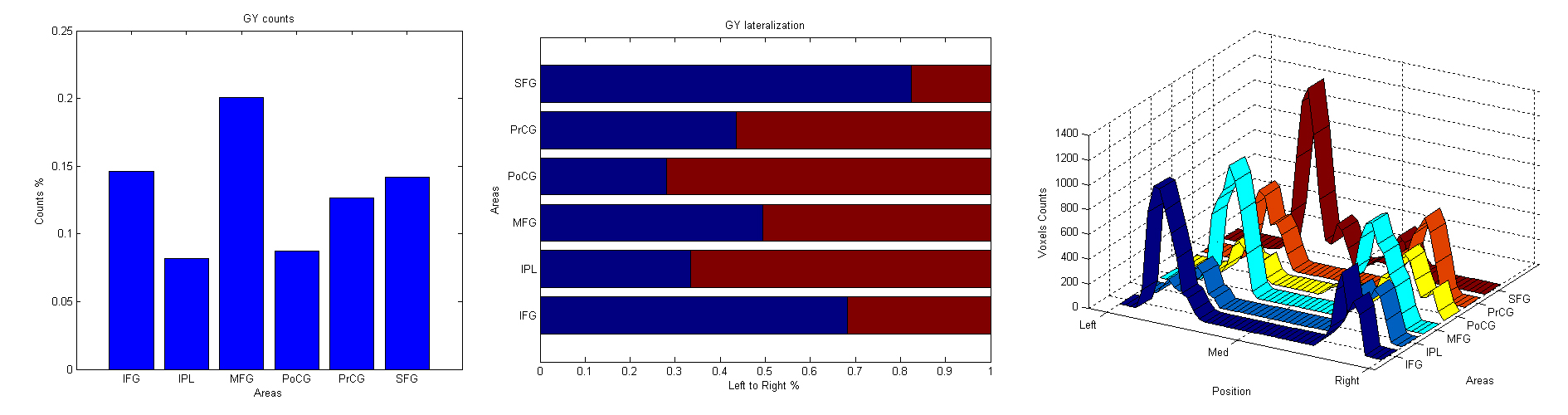
**

Most engaged gyri, from left to right: counts percent as function of areas, area lateralization (left blue, right red) and counts as function of area and X TAL coordinate; Abbreviations IFG = Inferior Frontal Gyrus, IPL = Inferior Parietal Lobule, MFG = Middle Frontal Gyrus, PoCG = Postcentral Gyrus, PrCG = Precentral Gyrus; SFG = Superior Frontal Gyrus; Two sample t-test q<0.05 FDR-corrected, minimum cluster dimension >5 voxels in the native resolution

**Fig s16: Activation distribution of the MDN rsFC increased connectivity**

**
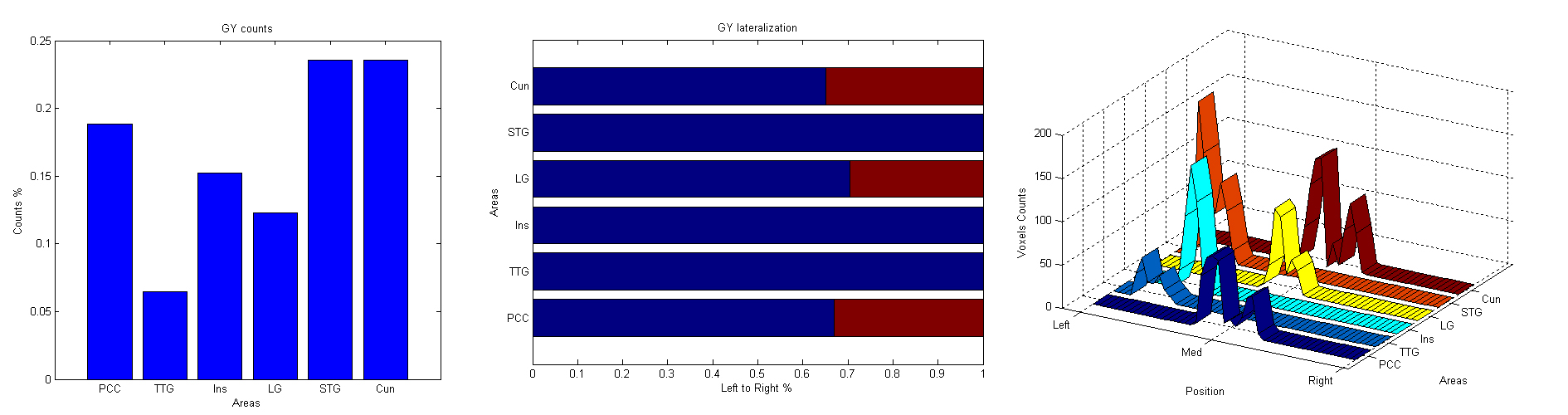
**

Most engaged gyri, from left to right: counts percent as function of areas, area lateralization (left blue, right red) and counts as function of area and X TAL coordinate; Abbreviations PCC = Posterior Cingulate Gyrus, TTG = Transverse Temporal Gyrus, Ins = Insula, LG = Lingual Gyrus, STG = Superior Temporal Gyrus, Cun = Cuneus; Two sample t-test q<0.05 FDR-corrected, minimum cluster dimension >5 voxels in the native resolution

**Fig s17: Activation distribution of the MDN rsFC decreased connectivity**

**
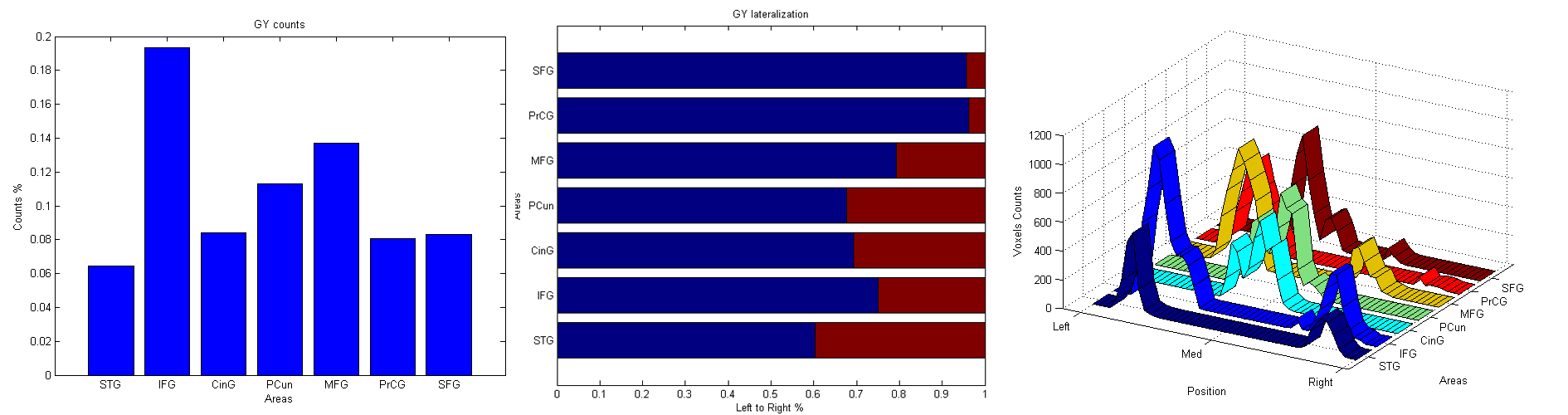
**

Most engaged gyri, from left to right: counts percent as function of areas, area lateralization (left blue, right red) and counts as function of area and X TAL coordinate; Abbreviations STG = Superior Temporal Gyrus, IFG = Inferior Frontal Gyrus, CingG = Cingulate Gyrus, PCun = Precuneus, MFG = Middle Frontal Gyrus, PrCG = Precentral Gyrus, MFG = Superior Frontal Gyrus; Two sample t-test q<0.05 FDR-corrected, minimum cluster dimension >5 voxels in the native resolution

**Fig s18: Voxel distance calculations**

**
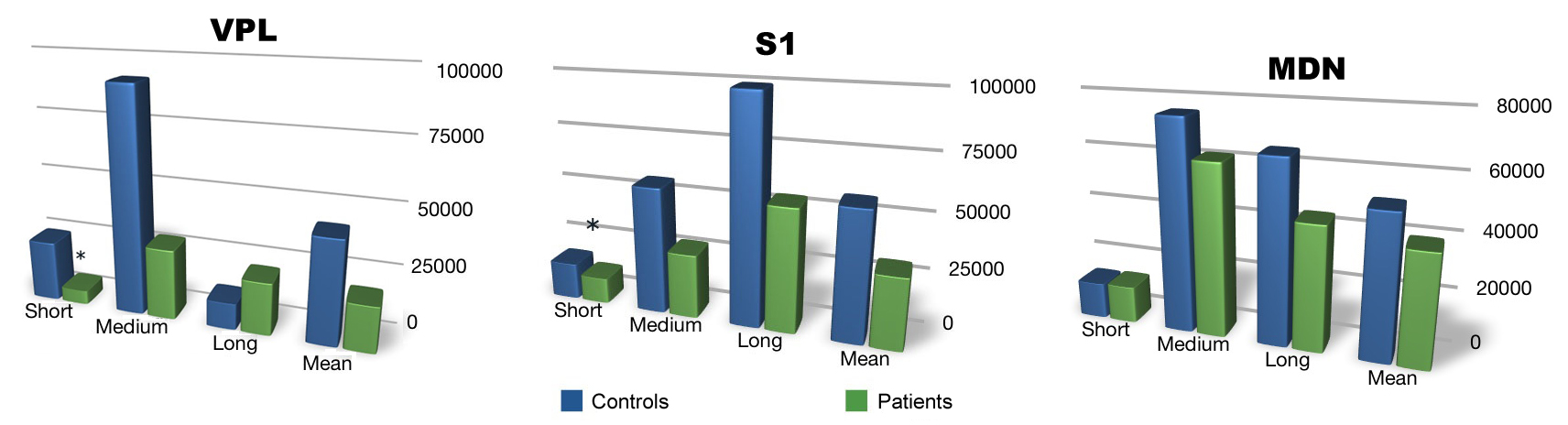
**

Mean distances between the seed ROI and every activated voxel divided into short (<40 mm), medium (40-80 mm), long (>80 mm) and mean (all) bins; In green the pain patients, in blue the control group, asterisk indicates significant differences between groups (t test p<0.05); From left to right VPL, S1 and MDN distances

**Fig s19: Functional connectivity stability**

Original S1 ROI and comparison with translated (3mm, rostral Ro, lateral La, ventral Ve, medial Me, caudal Ca) as well as increased (8x8x8 mm^3^) and decreased (3x3x3 mm^3^) ROI; On the extreme left of the figure the probabilistic map shows the spatial overlap of all eight different translated/increased/decreased ROI-generated maps

**Fig s20: Functional Connectivity stability**

**
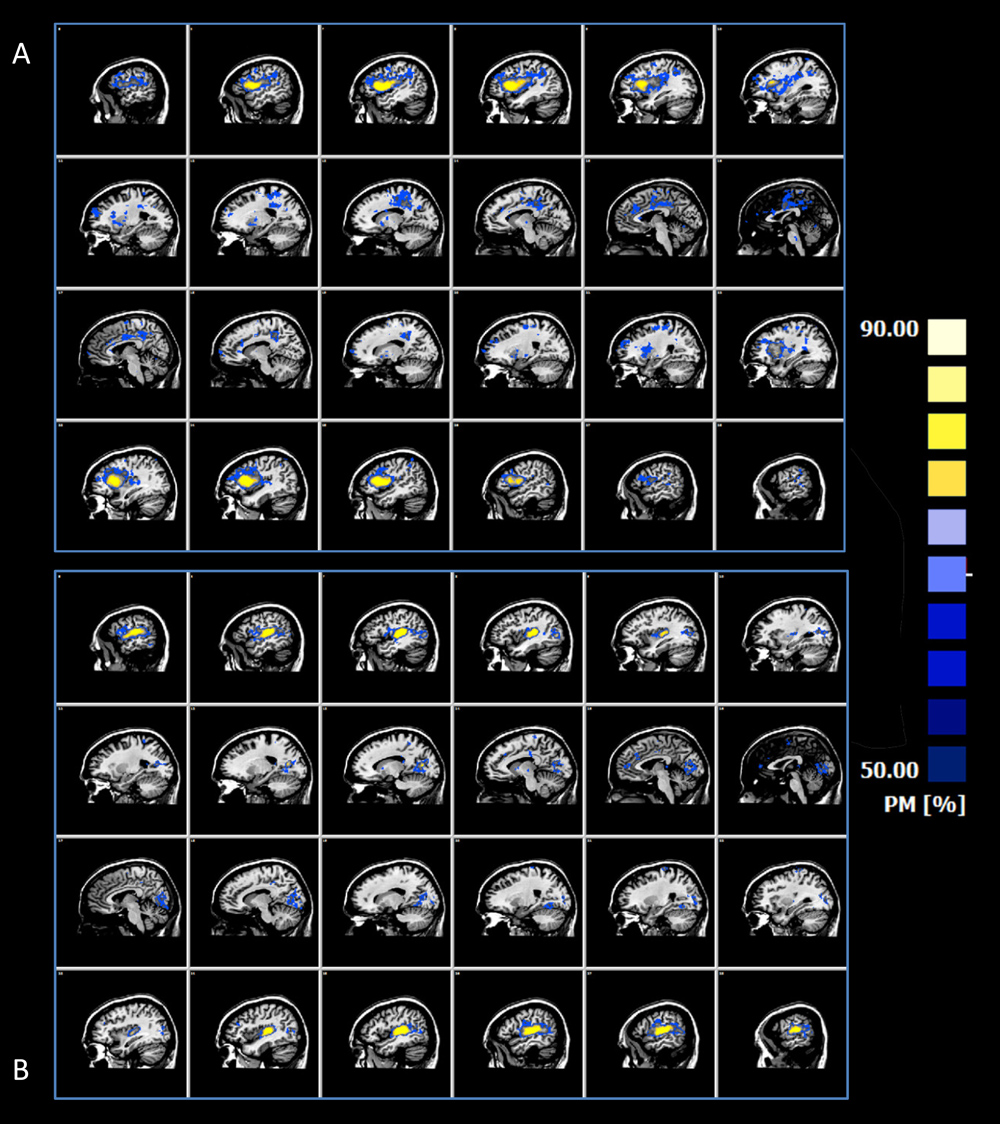
**

Probabilistic maps of specific clusters in linguistic (A) and auditory (B) networks rsFC relative to all subjects

Colors from blue to yellow indicate an increasing spatial overlapping probability (%)

Single subject correlation maps before probabilistic map creation thresholded at

q<0.05 FDR-corrected, cluster dimension >5 voxels in the native resolution

**Fig s21: Global Signal contribution**

**
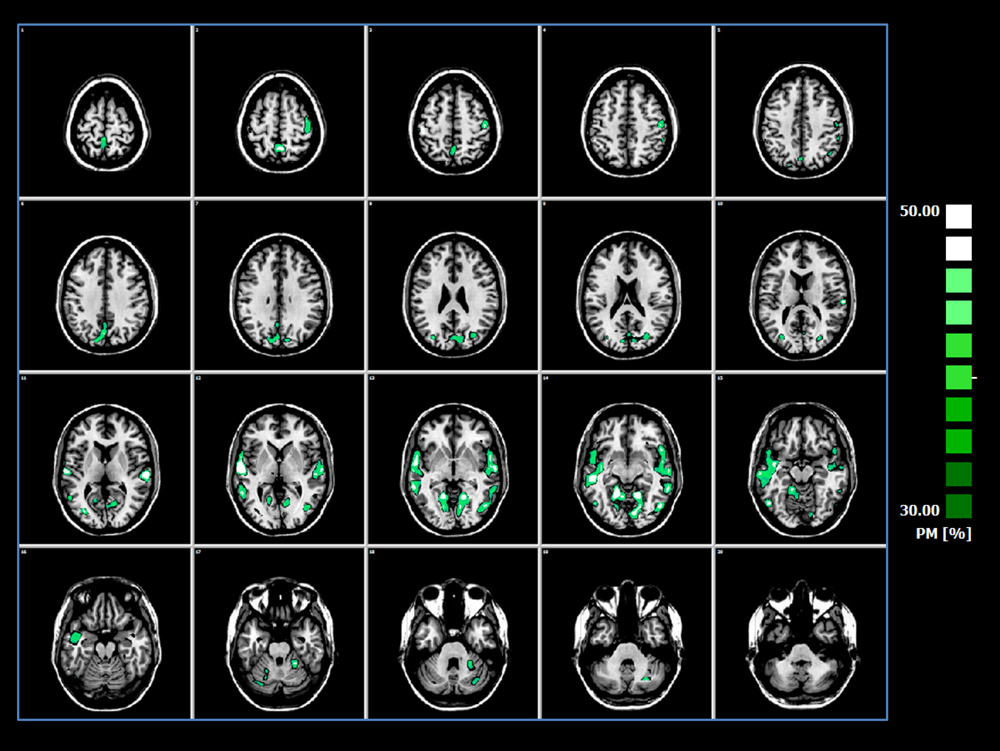
**

Probabilistic maps of rsFC voxel activity correlated to Global Signal relative to all subjects

Colors from green to white indicate an increasing spatial overlapping probability (%)

Single subject correlation maps before probability map creation thresholded at

q<0.05 FDR-corrected, cluster dimension >5 voxels in the native resolution

**Fig s22: S1 rsFC (all subjects)**

One sample t-test p<0.05 Bonferroni-corrected, minimum cluster dimension K>10 voxels in the native resolution

Maps projected on the 2D slices.

**Fig s23: VPL rsFC (all subjects)**

One sample t-test p<0.05 Bonferroni-corrected, minimum cluster dimension K>10 voxels in the native resolution

Maps projected on the 2D slices.

**Fig s24: MDN rsFC (all subjects)**

One sample t-test p<0.05 Bonferroni-corrected, minimum cluster dimension K>10 voxels in the native resolution

Maps projected on the 2D slices.

**Additional Tables**

**Table s1: Example of Activations Summary Output**

| Area | Voxels | L/R% | Left BA | Right BA |
| --- | --- | --- | --- | --- |
| Bilat Middle Frontal Gyrus | 27529 | 35/65% | 9 8 10 46 | 9 8 10 46 |
| Bilat Cingulate Gyrus | 26379 | 51/49% | 31 24 32 23 | 31 24 32 23 |
| Bilat Out of Gyrus | 16444 | 49/51% | 23 6 31 9 | 23 13 6 31 |
| Bilat Inferior Parietal Lobule | 13035 | 39/61% | 40 13 22 39 | 40 13 22 |
| Bilat Superior Frontal Gyrus | 8078 | 48/52% | 9 10 6 8 | 9 10 6 8 |
| Bilat Supramarginal Gyrus | 7561 | 33/67% | 40 39 | 40 39 |
| Bilat Inferior Frontal Gyrus | 4514 | 7/93% | 46 10 | 47 46 45 9 |
| Bilat Insula | 3279 | 1/99% | 13 40 | 13 47 22 21 |
| Bilat Precuneus | 3200 | 40/60% | 7 31 39 19 | 7 31 5 |
| Bilat Medial Frontal Gyrus | 2642 | 33/67% | 6 9 32 8 | 6 9 32 8 |
| Bilat Superior Temporal Gyrus | 2539 | 12/88% | 22 13 42 40 | 22 39 13 42 |
| Bilat Precentral Gyrus | 2503 | 24/76% | 9 6 4 8 | 9 6 44 |
| Bilat Paracentral Lobule | 1105 | 52/48% | 31 24 | 31 24 |
| Bilat Angular Gyrus | 833 | 42/58% | 39 40 | 39 40 |
| Bilat Posterior Cingulate | 724 | 50/50% | 23 | 23 |
| Bilat Anterior Cingulate | 698 | 65/35% | 32 24 | 32 24 |
| Right Cuneus | 360 | 0/100% |  | 7 18 19 31 |
| Right Ventral Lateral Nucleus | 319 | 0/100% |  |  |
| Bilat Postcentral Gyrus | 306 | 87/13% | 40 2 42 | 40 2 |
| Right Middle Temporal Gyrus | 214 | 0/100% |  | 21 20 39 |
| Right Medial Dorsal Nucleus | 159 | 0/100% |  |  |
| Right Ventral Posterior Lateral Nucleus | 137 | 0/100% |  |  |

**Table s2: Demographics of patients**

| **Patient** | **Pain duration (yrs)** | **Paresthesias** | **Dysesthesias** | **Quality of pain** | **VAS score**  **(0-10)** | **Distribution** |
| --- | --- | --- | --- | --- | --- | --- |
| **1** | 2 | + |  | Pulsating-burning | 6 | Lower limbs |
| **2** | 2 | + | + | Burning | 5 | Lower limbs |
| **3** | 3 |  | + | Burning-squeezing | 5 | Lower limbs |
| **4** | 3 | + | + | Burning-squeezing | 6 | Lower limbs |
| **5** | 2 | + |  | Lancinating-burning | 6 | Lower limbs |
| **6** | 2 | + |  | Burning-squeezing | 4 | Lower limbs |
| **7** | 3 |  | + | Burning-piercing | 9 | Lower limbs |
| **8** | 5 | + |  | Burning-piercing | 4 | Lower limbs |

**Table s3: ROI used as seed regions for rsFC analyses**

| **AREA** | **X** | **Y** | **Z** | **SIZE mm^3^** |
| --- | --- | --- | --- | --- |
| **S1 Left** | -43 | -26 | +49 | 125 |
| **S1 Right** | +46 | -22 | +49 | 125 |
| **VPL Left** | -18 | -20 | +5 | 125 |
| **VPL Right** | +20 | -20 | +5 | 125 |
| **MDN Left** | -6 | -22 | +8 | 125 |
| **MDN Right** | +6 | -22 | +8 | 125 |

**Tab s4: S1 rsFC group comparison: increased connectivity in the pain group**

| Area | Voxels | L/R% | Left BA | Right BA |
| --- | --- | --- | --- | --- |
| Bilat Inferior Parietal Lobule | 9242 | 31/69% | 40 7 2 | 40 7 39 |
| Bilat Precuneus | 6509 | 70/30% | 7 31 | 7 19 39 31 |
| Bilat Out of Gyrus | 3980 | 24/76% | 40 7 31 | 40 39 7 19 |
| Bilat Postcentral Gyrus | 2786 | 33/67% | 2 3 1 40 | 2 3 1 40 |
| Bilat Precentral Gyrus | 2213 | 1/99% | 4 2 | 6 4 9 |
| Bilat Supramarginal Gyrus | 1256 | 19/81% | 40 | 40 |
| Bilat Superior Parietal Lobule | 972 | 77/23% | 7 | 7 19 |
| Right Cuneus | 522 | 0/100% |  | 19 7 18 31 |
| Bilat Angular Gyrus | 382 | 3/97% | 40 | 39 40 19 |
| Left Cingulate Gyrus | 318 | 100/0% | 31 |  |

Thresholds: Total activation voxels 5%; Cortical Area activation voxels 25%; Subcortical Area activation voxels 125

**Tab s5: S1 rsFC group comparison: decreased connectivity in the pain group**

| Area | Voxels | L/R% | Left BA | Right BA |
| --- | --- | --- | --- | --- |
| Bilat Postcentral Gyrus | 5101 | 64/36% | 3 2 40 43 | 3 2 40 |
| Bilat Out of Gyrus | 4454 | 46/54% | 18 19 3 | 18 13 19 24 |
| Bilat Middle Temporal Gyrus | 4375 | 54/46% | 22 21 37 39 | 22 21 |
| Bilat Superior Temporal Gyrus | 4373 | 72/28% | 22 21 42 41 | 22 21 42 41 |
| Bilat Precentral Gyrus | 2340 | 85/15% | 4 6 3 43 | 4 6 |
| Bilat Middle Occipital Gyrus | 1331 | 67/33% | 18 19 37 | 18 |
| Bilat Inferior Parietal Lobule | 1019 | 3/97% | 40 2 13 | 40 2 13 |
| Bilat Middle Frontal Gyrus | 888 | 14/86% | 9 8 4 | 9 46 |
| Bilat Paracentral Lobule | 821 | 11/89% | 5 6 31 | 5 6 31 |
| Right Putamen | 764 | 0/100% |  |  |
| Right Declive | 707 | 0/100% |  | 19 18 |
| Bilat Medial Frontal Gyrus | 685 | 24/76% | 6 9 | 6 31 |
| Left Superior Frontal Gyrus | 631 | 100/0% | 9 10 |  |
| Bilat Cingulate Gyrus | 535 | 63/37% | 24 32 | 24 |
| Bilat Inferior Occipital Gyrus | 445 | 89/11% | 19 18 | 18 |
| Bilat Cuneus | 354 | 1/99% |  | 17 30 18 |
| Bilat Fusiform Gyrus | 279 | 0/100% | 19 | 19 37 |
| Bilat Lingual Gyrus | 222 | 52/48% | 18 19 | 18 19 |

Thresholds: Total activation voxels 5%; Cortical Area activation voxels 25%; Subcortical Area activation voxels 125

**Tab s6: S1 rsFC group comparison: increased and decreased connectivity in the pain group cluster by cluster**

Thresholds: Total activation voxels 5%; Cortical Area activation voxels 25%; Subcortical Area activation voxels 125

**Tab s7: VPL rsFC group comparison: increased connectivity in the pain group**

| Area | Voxels | L/R% | Left BA | Right BA |
| --- | --- | --- | --- | --- |
| Bilat Lingual Gyrus | 1451 | 91/9% | 18 19 | 18 |
| Bilat Cuneus | 860 | 86/14% | 30 18 23 17 | 30 18 |
| Bilat Out of Gyrus | 257 | 48/52% | 18 30 | 18 30 |
| Bilat Posterior Cingulate | 247 | 92/8% | 30 31 23 | 30 |

Thresholds: Total activation voxels 5%; Cortical Area activation voxels 25%; Subcortical Area activation voxels 125

**Tab s8: VPL rsFC group comparison: decreased connectivity in the pain group**

| Area | Voxels | L/R% | Left BA | Right BA |
| --- | --- | --- | --- | --- |
| Bilat Out of Gyrus | 13623 | 45/55% | 10 13 6 9 | 10 13 6 9 |
| Bilat Middle Frontal Gyrus | 10807 | 50/50% | 10 9 46 6 | 10 9 46 6 |
| Bilat Inferior Frontal Gyrus | 7876 | 68/32% | 45 46 47 13 | 45 46 9 |
| Bilat Superior Frontal Gyrus | 7657 | 82/18% | 10 9 6 8 | 10 9 6 |
| Bilat Precentral Gyrus | 6836 | 44/56% | 6 4 44 3 | 6 4 44 43 |
| Bilat Postcentral Gyrus | 4713 | 28/72% | 3 2 5 | 3 2 5 7 |
| Bilat Inferior Parietal Lobule | 4408 | 33/67% | 40 7 13 | 40 7 2 13 |
| Bilat Cingulate Gyrus | 2689 | 40/60% | 32 24 31 | 32 24 |
| Bilat Insula | 2434 | 67/33% | 13 44 47 45 | 13 44 6 |
| Bilat Precuneus | 1595 | 0/100% | 7 | 7 |
| Bilat Medial Frontal Gyrus | 1252 | 70/30% | 10 9 6 | 10 9 6 |
| Bilat Supramarginal Gyrus | 926 | 82/18% | 40 39 | 40 |
| Bilat Superior Parietal Lobule | 790 | 10/90% | 7 5 | 7 40 |
| Bilat Anterior Cingulate | 753 | 89/11% | 24 10 32 33 | 24 10 32 |
| Right Middle Temporal Gyrus | 740 | 0/100% |  | 21 22 37 20 |
| Right Caudate Body | 653 | 0/100% |  |  |
| Bilat Superior Temporal Gyrus | 217 | 47/53% | 42 22 39 40 | 42 22 40 13 |
| Left Putamen | 213 | 100/0% |  |  |
| Left Pulvinar | 202 | 100/0% |  |  |
| Left Medial Dorsal Nucleus | 133 | 100/0% |  |  |

Thresholds: Total activation voxels 5%; Cortical Area activation voxels 25%; Subcortical Area activation voxels 125

**Tab s9: VPL rsFC group comparison: increased and decreased connectivity in the pain group cluster by cluster**

Thresholds: Total activation voxels 5%; Cortical Area activation voxels 25%; Subcortical Area activation voxels 125

**Tab s10: MDN rsFC group comparison: increased connectivity in the pain group**

| Area | Voxels | L/R% | Left BA | Right BA |
| --- | --- | --- | --- | --- |
| Left Superior Temporal Gyrus | 579 | 100/0% | 41 13 22 42 |  |
| Bilat Cuneus | 579 | 65/35% | 30 18 23 | 30 18 |
| Bilat Posterior Cingulate | 463 | 67/33% | 30 31 23 | 30 |
| Bilat Out of Gyrus | 385 | 72/28% | 18 30 13 | 18 30 |
| Left Insula | 374 | 100/0% | 13 |  |
| Bilat Lingual Gyrus | 301 | 70/30% | 18 | 18 |
| Left Transverse Temporal Gyrus | 159 | 100/0% | 41 13 42 |  |

Thresholds: Total activation voxels 5%; Cortical Area activation voxels 25%; Subcortical Area activation voxels 125

**Tab s11: MDN rsFC group comparison: decreased connectivity in the pain group**

| Area | Voxels | L/R% | Left BA | Right BA |
| --- | --- | --- | --- | --- |
| Bilat Out of Gyrus | 13551 | 83/17% | 21 6 10 | 10 34 22 |
| Bilat Inferior Frontal Gyrus | 7569 | 75/25% | 47 45 46 44 | 47 45 46 |
| Bilat Middle Frontal Gyrus | 5359 | 79/21% | 10 6 11 47 | 10 11 47 |
| Bilat Precuneus | 4412 | 68/32% | 7 31 | 7 31 |
| Bilat Cingulate Gyrus | 3280 | 69/31% | 31 24 6 | 31 24 6 |
| Bilat Superior Frontal Gyrus | 3257 | 96/4% | 10 9 6 8 | 10 11 |
| Bilat Precentral Gyrus | 3146 | 96/4% | 6 4 44 3 | 4 44 3 45 |
| Bilat Superior Temporal Gyrus | 2520 | 60/40% | 22 38 13 | 22 38 47 |
| Bilat Postcentral Gyrus | 1773 | 51/49% | 2 3 40 4 | 2 3 40 |
| Bilat Pulvinar | 1337 | 83/17% |  |  |
| Bilat Medial Frontal Gyrus | 972 | 52/48% | 6 10 9 | 6 10 24 |
| Bilat Paracentral Lobule | 910 | 68/32% | 31 5 6 | 31 6 |
| Bilat Parahippocampal Gyrus | 793 | 46/54% | 34 27 28 30 | 34 28 |
| Bilat Anterior Cingulate | 715 | 58/42% | 25 10 33 | 25 10 32 |
| Right Pyramis | 711 | 0/100% |  |  |
| Bilat Inferior Parietal Lobule | 685 | 52/48% | 40 2 | 40 2 |
| Right Uvula | 685 | 0/100% |  |  |
| Left Supramarginal Gyrus | 513 | 100/0% | 40 |  |
| Left Ventral Posterior Lat Nucleus | 463 | 100/0% |  |  |
| Bilat Amygdala | 393 | 60/40% |  |  |
| Left Lateral Globus Pallidus | 374 | 100/0% |  |  |
| Bilat Insula | 362 | 64/36% | 13 22 47 | 13 22 47 38 |
| Bilat Cerebellar Tonsil | 352 | 7/93% |  |  |
| Right Nodule | 220 | 0/100% |  |  |
| Left Hypothalamus | 206 | 100/0% |  |  |
| Left Hippocampus | 196 | 100/0% |  |  |
| Bilat Subcallosal Gyrus | 163 | 5/95% | 25 | 34 25 47 |
| Left Subthalamic Nucleus | 159 | 100/0% |  |  |
| Left Ventral Lateral Nucleus | 141 | 100/0% |  |  |
| Left Caudate Tail | 133 | 100/0% |  |  |

**Tab s12: MDN rsFC group comparison: increased and decreased connectivity in the pain group cluster by cluster**

Thresholds: Total activation voxels 5%; Cortical Area activation voxels 25%; Subcortical Area activation voxels 125
